# Metal–carbon bonding in early lanthanide substituted cyclopentadienyl complexes probed by pulsed EPR spectroscopy†

**DOI:** 10.1039/d3sc06175b

**Published:** 2024-01-16

**Authors:** Lydia E. Nodaraki, Jingjing Liu, Ana-Maria Ariciu, Fabrizio Ortu, Meagan S. Oakley, Letitia Birnoschi, Gemma K. Gransbury, Philip J. Cobb, Jack Emerson-King, Nicholas F. Chilton, David P. Mills, Eric J. L. McInnes, Floriana Tuna

**Affiliations:** a Department of Chemistry, The University of Manchester Oxford Road Manchester M13 9PL UK floriana.tuna@manchester.ac.uk nicholas.chilton@manchester.ac.uk david.mills@manchester.ac.uk eric.mcinnes@manchester.ac.uk; b Photon Science Institute, The University of Manchester Oxford Road Manchester M13 9PL UK

## Abstract

We examine lanthanide (Ln)–ligand bonding in a family of early Ln^3+^ complexes [Ln(Cp^tt^)_3_] (1-Ln, Ln = La, Ce, Nd, Sm; Cp^tt^ = C_5_H_3_^*t*^Bu_2_-1,3) by pulsed electron paramagnetic resonance (EPR) methods, and provide the first characterization of 1-La and 1-Nd by single crystal XRD, multinuclear NMR, IR and UV/Vis/NIR spectroscopy. We measure electron spin *T*_1_ and *T*_m_ relaxation times of 12 and 0.2 μs (1-Nd), 89 and 1 μs (1-Ce) and 150 and 1.7 μs (1-Sm), respectively, at 5 K: the *T*_1_ relaxation of 1-Nd is more than 10^2^ times faster than its valence isoelectronic uranium analogue. ^13^C and ^1^H hyperfine sublevel correlation (HYSCORE) spectroscopy reveals that the extent of covalency is negligible in these Ln compounds, with much smaller hyperfine interactions than observed for equivalent actinide (Th and U) complexes. This is corroborated by *ab initio* calculations, confirming the predominant electrostatic nature of the metal–ligand bonding in these complexes.

## Abstract 2

We report relaxation and ligand hyperfine effects in an isostructural series of early lanthanide substituted cyclopentadienyl complexes, [Ln(Cp^tt^)_3_] (Ln = Ce, Nd, Sm; Cp^tt^ = C_5_H_3_^*t*^Bu_2_-1,3), by pulsed EPR spectroscopy.

## Graphical abstract

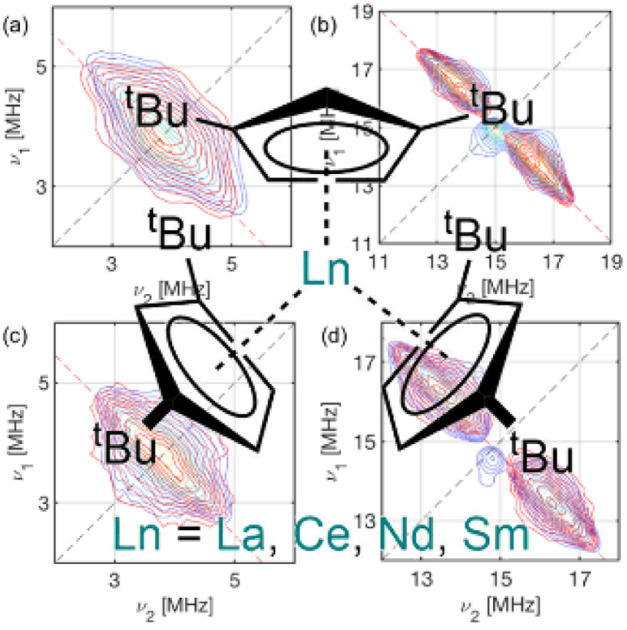

## Introduction

Lanthanides (Ln) have numerous applications due to their unique physicochemical properties.^1^ The text-book description of the electronic structure of Ln ions in the dominant 3+ oxidation state is that due to poor shielding the valence 4f orbitals are “core-like,” and as a result chemical bonding involving Ln ions is predominantly ionic. In contrast to Ln, the early actinides (An) exhibit variable oxidation states and participation in metal–ligand multiple bonding, which indicates greater involvement of the 5f (and 6d) valence orbitals in bonding regimes.^2^ This is important because the differences in chemical bonding has direct impacts on the chemical behavior of Ln and An ions,^3^ and such differences are exploited in, for example, separations chemistry.^4,5^ Moreover, subtle variations in f-element-ligand bonding is important in determining electronic properties and hence applications in, for example, luminescence,^6^ paramagnetic NMR,^7^ and molecular magnetism.^8^ Use of relatively soft donor ligands, such as substituted cyclopentadienyls (Cp^R^), can introduce interesting bonding regimes, and their Ln/An complexes have been studied for many years.^9–11^ Such materials have found interest not just in chemical synthesis, but also due to their electronic structure leading to, for example, the stabilization of unprecedented metal oxidation states^12^ and properties of single-molecule magnets.^13^ The archetypal examples are the [Ln/An(Cp)_3_] type complexes, and indeed these have long been used as a computational test-bed to investigate trends in the nature of f-element bonding.^14–18^

Since the majority of Ln^3+^ and An^3+^ ions are paramagnetic, electron paramagnetic resonance (EPR) spectroscopy should carry important information on metal–ligand bonding, particularly if resolution of ligand nuclear hyperfine interactions can be resolved. However, this is rarely the case in continuous wave (CW) EPR because of the intrinsically broad linewidths. This can in principle be addressed by pulsed EPR hyperfine methods.^19^ However, there are surprisingly few pulsed EPR studies on molecular Ln complexes,^20^ with the notable exception of those of the ^8^S_7/2_ Gd^3+^ ion, and even fewer for An complexes.^21–23^ Most Ln^3+^ pulsed EPR studies have been performed on doped minerals or glasses, and have tended to focus on relaxation behaviour.^24^ In 2011 Denning and co-workers reported pulsed EPR measurements on [Yb(Cp)_3_], enabling quantification of the significant spin density at the ^13^C atoms of the Cp rings,^25^ which was discussed in terms of mixing of the ^2^F_7/2_ ground term with low-lying charge-transfer states. More recently, some of us reported pulsed EPR studies of two An complexes, [An(Cp^tt^)_3_] (1-An; An = Th, U; Cp^tt^ = C_5_H_3_^*t*^Bu_2_-1,3), again showing significant spin density on the Cp^tt^ ligands for both the U^3+^ and Th^3+^ analogues.^21^

Here we report studies, including pulsed EPR, on a family of early Ln^3+^ complexes [Ln(Cp^tt^)_3_] (1-Ln, Ln = La, Ce, Nd, Sm). This allows: (i) investigation of trends in metal–arene interactions across the series, from the “parent” diamagnetic, f^0^ complex through the paramagnetic f^1,3,5^ analogues. The latter are chosen because they are all Kramers ions and hence are expected to be EPR-active; (ii) comparison of ligand spin density with the late Ln^3+^ complex [Yb(Cp)_3_] studied by Denning *et al.*;^25^ (iii) comparision of 1-Ln with the early An^3+^ homologues 1-An.^21^ The latter includes direct comparison of the valence isoelectronic Nd^3+^ (4f^3^) and U^3+^ (5f^3^) pair, which is the only comparison available between 4f and 5f M^3+^ ions that does not require designated radiochemical laboratories (although Ce^3+^ and Th^3+^ have the same number of valence electrons, the Th^3+^ ion often favours a 6d^1^ rather than 5f^1^ configuration).^26^

Complexes 1-Ln are readily prepared by reacting LnCl_3_ with three equivalents of KCp^tt^; 1-Ce ^27^ and 1-Sm ^28^ have been prepared previously by alternative synthetic routes, whilst 1-La and 1-Nd are structurally characterised here for the first time. We report the CW and pulsed EPR data for 1-Ce, 1-Nd and 1-Sm, along with the NMR data of diamagnetic 1-La. We quantify the hyperfine interaction in paramagnetic 1-Ln with ligand nuclei, originating from interaction with the Ln^3+^ centres, and show that we can model these data using a simple point-dipole model. This is in agreement with fully-*ab initio* complete active space self-consistent field spin–orbit (CASSCF-SO) calculations that directly report hyperfine coupling parameters,^29^ as well as spin densities. We find, using either the simple or *ab initio* methods, that there is negligible spin density on the Cp^tt^ ligands in 1-Ln, in contrast to unequivocal spin delocalisation in U^3+^ and Th^3+^ analogues [An(Cp^tt^)_3_],^21^ clearly highlighting the differences between early 4f and early 5f bonding in *tris*-Cp complexes.^14–18^ Since the ability to perform such studies are limited by electron spin relaxation times, we also report relaxation data for 1-Ln by pulsed EPR methods. We find that 4f 1-Ln relax orders of magnitude faster than their 5f An analogues,^21^ despite preconceptions that relaxation rates increase for heavier elements due to increased spin–orbit coupling.

## Results and discussion

### Synthesis

**Scheme 1:**
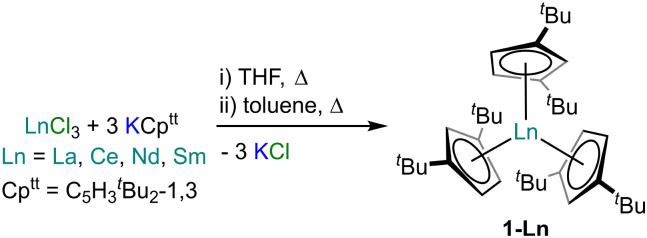
Synthesis of 1-Ln (Ln = La, Ce, Nd, Sm).

Complexes 1-Ln (Ln = La, Ce, Nd, Sm) were prepared from the parent LnCl_3_ and three equivalents of KCp^tt^ (Scheme 1) by modification of the reported syntheses of 1-Sm using SmI_3_ and KCp^tt^,^28^ and 1-Ce from Ce(OTf)_3_ and LiCp^tt^.^27^ The crystalline yields for 1-La, 1-Ce, 1-Nd and 1-Sm were 41%, 54%, 34% and 52%, respectively. We attempted to extend the 1-Ln series to smaller Ln, but in our hands we found that applying the same synthetic methodology to GdCl_3_ gave no isolable products, likely due to the higher Lewis acidity of the smaller Ln^3+^ ions.^1^ NMR spectroscopy was used to analyse C_6_D_6_ solutions of 1-Ln (ESI, Fig. S1–S5†). ^1^H NMR spectra were fully assigned for all 1-Ln; in each case three signals were observed in a ratio of 54 : 6 : 3 that correspond to the ^*t*^Bu groups and the two unique environments of the Cp^tt^ ring protons, respectively. The paramagnetism of 1-Ce, 1-Nd and 1-Sm precluded assignment of their ^13^C{^1^H} NMR spectra, however, for diamagnetic 1-La this could be interpreted, with the two ^*t*^Bu group resonances seen at 32.77 and 33.75 ppm and the three Cp^tt^ ring carbon environments found at 110.57 (*C*H-Cp), 110.69 (*C*H-Cp) and 143.45 (*C*-Cp) ppm. Although NMR spectroscopy showed few protic impurities, elemental analysis results for 1-Ln consistently gave low carbon values; this was previously seen for [An(Cp^tt^)_3_],^21^ and is ascribed to carbide formation leading to partial combustion, a common issue with these experiments.^30^ In the case of 1-Sm elemental analysis results were not in accord with expected values. However, microcrystalline samples of 1-Ln gave essentially superimposable ATR-IR spectra (ESI, Fig. S6–S10†), and when these samples were suspended in a minimum amount of fomblin they gave poorly resolved powder XRD patterns (Fig. S13–S16†) that are similar to each other and show peaks in common with predicted patterns from single crystal XRD data (see below). Together, the characterisation data obtained show the bulk purities of samples of 1-Ln.

### Crystallography

**Fig. 1:**
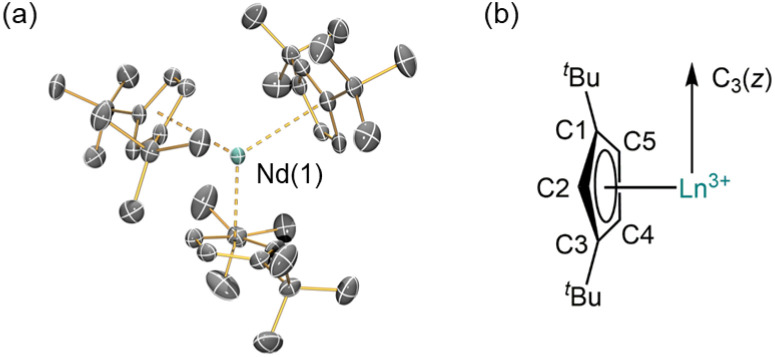
(a) Crystal structure of 1-Nd; Nd = cyan, C = grey. Displacement ellipsoids are at the 30% probability level. Hydrogen atoms are omitted for clarity. (b) Schematic of *C*_3_ symmetry of the structures of 1-Ln.

The solid-state structures of 1-Ln were determined by single crystal XRD (1-Nd is depicted in Fig. 1, see ESI, Fig. S11 and S12† for selected distances and angles of 1-La and 1-Nd). The structural data of 1-La and 1-Nd are reported here for the first time, whilst 1-Ce ^27^ and 1-Sm ^28^ have been reported previously; although 1-Ln are isostructural they are not all isomorphic. As expected, the structures of 1-Ln are trigonal planar with respect to the *η*^5^-Cp^tt^ centroids, with the three C2 atoms (Fig. 1) in the plane defined by the Ln^3+^ ion and the three Cp^tt^ centroids. The three Cp^tt^ ligands adopt the same orientation to form a “picket-fence” motif with three ^*t*^Bu groups above and three below the trigonal plane. Complexes 1-Ln do not show high symmetry in the solid state, consistent with the solid-state structures of [M(Cp^tt^)_3_] (M = Th,^21^ U,^21^ Yb^31^). The mean Ln⋯Cp_centroid_ distances decrease regularly across the lanthanide series [mean La⋯Cp_centroid_ = 2.635 Å; Ce⋯Cp_centroid_ = 2.596 Å;^27^ Nd⋯Cp_centroid_ = 2.56 Å; Sm⋯Cp_centroid_ = 2.531 Å;^28^ Yb⋯Cp_centroid_ = 2.47 Å^31^]. Additionally, the mean M⋯Cp_centroid_ distances in 1-Nd are shorter than those seen in [U(Cp^tt^)_3_] [2.570 Å],^21^ which has an analogous electronic configuration (Nd^3+^: [Xe]4f^3^; U^3+^: [Rn]5f^3^). Conversely, in 1-Ce, the mean M⋯Cp_centroid_ distances are longer than those seen in [Th(Cp^tt^)_3_] [2.566 Å],^21^ which has a different valence electronic configuration (Ce^3+^: [Xe]4f^1^; Th^3+^: [Rn]6d^1^) due to the near-degeneracy of the 5f and 6d orbitals for Th^2^ and the stabilization of the 6d_*z*^2^_ orbital in trigonal ligand environments.^26,32^

### Solution phase optical properties

**Fig. 2:**
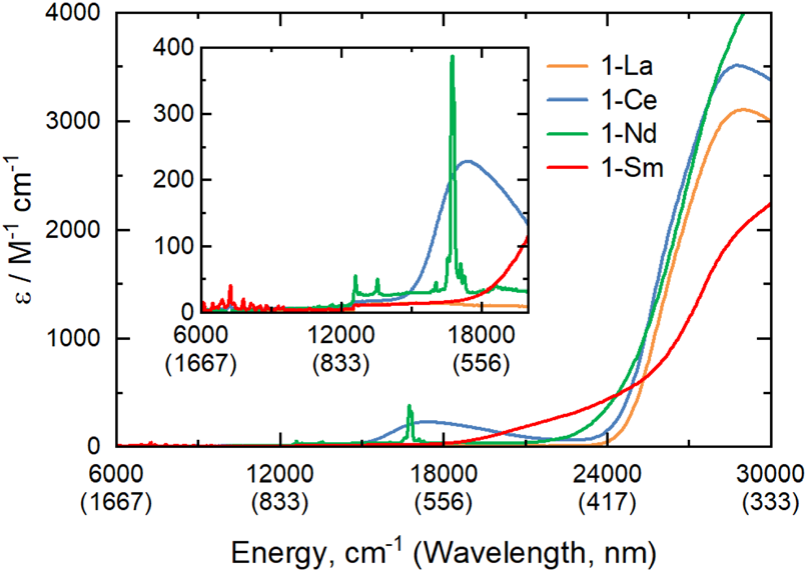
UV/vis/NIR spectra of 1-La, 1-Ce, 1-Nd, 1-Sm, from 6000–30 000 cm^−1^ (inset 6000–20 000 cm^−1^) recorded as 0.5 mM solutions in toluene at room temperature.

The electronic spectra of 0.5 mM toluene solutions of 1-Ln (Fig. 2 and ESI, Fig. S17–S21†) are as expected;^1^1-La with a closed shell La^3+^ ion is essentially colourless, with charge-transfer (CT) bands restricted to the UV region, whilst 1-Ce, 1-Nd and 1-Sm are pale purple, pale green, and pale orange, respectively. Due to their Laporte-forbidden nature, even spin-allowed f–f transitions are relatively weak (*ε* < 400 mol^−1^ dm^3^ cm^−1^),^1^ so CT bands tailing in from the UV region dominate the spectra. The f–f transitions in 1-Nd were clearly observed, with the most intense absorption at υ̃_max_ = 16 750 cm^−1^ (*ε* = 390 mol^−1^ dm^3^ cm^−1^) likely arising from the ^4^I_9/2_ → ^4^G_5/2_/^4^G_7/2_ transition.^33^ In contrast, 1-Ce shows a strong broad absorption at υ̃_max_ = 17 400 cm^−1^ (*ε* = 230 mol^−1^ dm^3^ cm^−1^) due to [Xe]4f^1^ → [Xe]4f^0^5d^1^ transitions, which are formally allowed by electric dipole selection rules. The electronic spectrum of 1-Sm shows a weak set of f–f transitions in the near-IR region around 7000 cm^−1^ (*ε* < 50 mol^−1^ dm^3^ cm^−1^), likely arising from the ^6^H_5/2_ → ^6^F_*J*_ transitions.^33^

### Magnetism

**Fig. 3:**
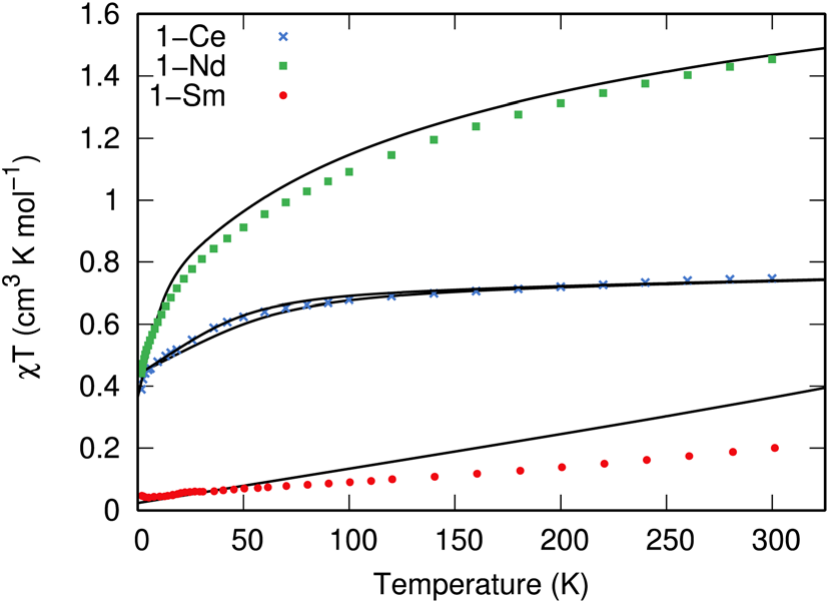
Temperature dependence of *χT* for 1-Nd (green squares), 1-Ce (blue crosses), and 1-Sm (red circles) recorded at 0.1, 0.1 and 0.5 T. Calculated temperature dependence of *χT* using CASSCF-SO is given with black lines using the XRD structure for each molecule; two traces for 1-Ce arise from each of the non-equivalent molecules in the crystal structure.

The measured *χT* values of 1.45 and 0.75 cm^3^ mol^−1^ K at 300 K for 1-Nd and 1-Ce, respectively (Fig. 3), are close to the expected values of 1.63 and 0.80 cm^3^ mol^−1^ K for Nd^3+^ (^4^I_9/2_) and Ce^3+^ (^2^F_5/2_).^1^ The *χT* product decreases gradually upon cooling until below 50 or 60 K, where it drops to reach 0.44 and 0.30 cm^3^ mol^−1^ K at 1.8 K, respectively. For 1-Sm, *χT* is 0.20 cm^3^ mol^−1^ K at 300 K, which is larger than expected for an isolated free-ion ^6^H_5/2_ term of 0.09 cm^3^ mol^−1^ K, owing to a combination of thermal population of the low-lying ^6^H_7/2_ term and temperature independent paramagnetism. The decrease of *χT* in all cases is due to the depopulation of the crystal field states. Isothermal magnetisation measurements of all compounds deviate from values expected for pure Ising-like *m*_J_ states (ESI, Fig. S22–S24†),^34^ indicating significant mixing of *m*_J_ states. To corroborate these results, we performed CASSCF-SO calculations using a minimal *n*-electrons in 7 orbital active space (where *n* = 1 for Ce^3+^, *n* = 3 for Nd^3+^ and *n* = 5 for Sm^3+^; see ESI† for details) and the solid-state XRD geometries (Tables S10–S13†). These give excellent reproductions of the magnetic data (Fig. 3 and S22–S24†) and confirm substantially mixed *m*_J_ states (Tables S10–S13†); the ground Kramers doublet for 1-Ce is dominated by |*m*_J_| = 1/2, with the first excited state at 105–135 cm^−1^, while for 1-Nd the ground Kramers doublet is dominated by *m*_J_ = ±5/2 mixed with ∓7/2, with a first excited state at 26 cm^−1^. For 1-Sm the ground Kramers doublet is dominated by *m*_J_ = ±3/2, with a first excited state at 400 cm^−1^.

### EPR spectroscopy

**Fig. 4:**
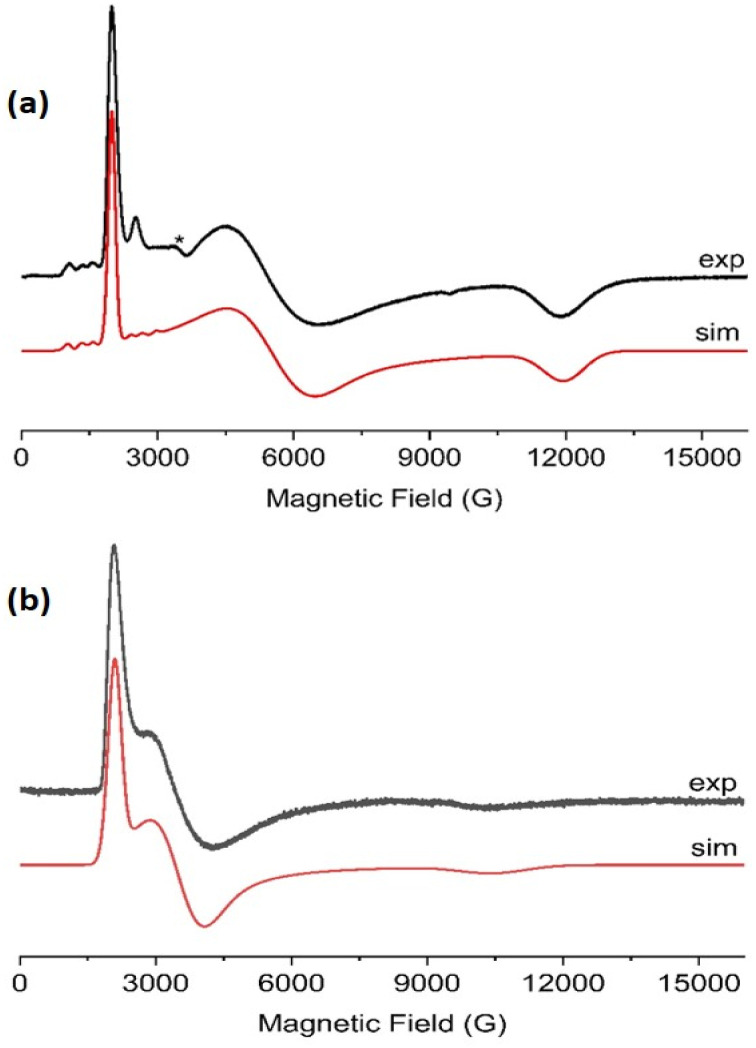
X-Band CW EPR spectra, frozen toluene : hexane (9 : 1) solutions of (a) 1-Nd at 8 K, and (b) 1-Ce at 10 K. The resonator cavity is marked with (*), and the simulation parameters are provided in the text and Table S2.

CW EPR spectra of 1-Nd, 1-Ce and 1-Sm are observed at temperatures below ∼30 K (Fig. 4 and ESI, Fig. S25–S27†). Much sharper spectra are observed from powders (such that it is difficult to remove polycrystalline effects) than from frozen solutions (toluene/hexane 9 : 1 v/v), indicating some relaxation and strain of the structures in the latter medium. The spectra are dominated in each case by the rhombic or axial effective *g*-values arising from transitions within a thermally isolated ground Kramers doublet of the ground Russell-Saunders state, which can be treated as an effective spin-1/2. In this approximation, the frozen solution EPR spectrum for 1-Nd (Fig. 4a) can be modeled with the spin Hamiltonian eqn (1).1
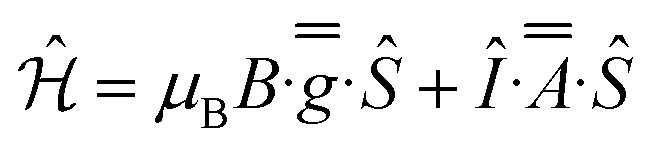


This gives *g*_*x*,*y*,*z*_ = 3.33, 1.22, 0.56, and hyperfine coupling constants of *A*_*x*_ = 1280 MHz (*A*_*y*_ and *A*_*z*_ unresolved; we have assumed *g̿* and *A̿* are collinear) to the *I* = 7/2 nuclear spin of Nd (natural abundance: 12.2% ^143^Nd and 8.3% ^145^Nd). Similarly, frozen solution spectra of 1-Ce and 1-Sm can be simulated with *g*_*x*,*y*,*z*_ = 3.15, 1.88, 0.64 for 1-Ce (Fig. 4b; note there are no isotopes of Ce with *I* ≠ 0), and *g*_*x*_ = *g*_*y*_ = 0.92 for 1-Sm (Fig. S27†). The *g*_*z*_ component of 1-Sm is not well resolved in CW spectra but is more clearly defined in pulsed spectra giving *g*_*z*_ = 0.45 (Fig. S28;† see below). CW spectra of the related [Nd(Cp′′)_3_] (Cp′′ = C_5_H_3_-(SiMe_3_)_2_-1,3) have been reported,^35^ and although the precise *g*-values differ the pattern is similar.

For 1-Ce and 1-Nd, CASSCF-SO calculations (Tables S10–S13; † note there are two non-equivalent molecules in the XRD structure of 1-Ce) give effective *g*-values for the lowest Kramers doublets as *g*_*x*,*y*,*z*_ = 2.1, 3.0, 0.7 and 2.2, 2.4, 1.1, respectively, with the numerically smallest value (*g*_*z*_) corresponding to the orientation of the pseudo-C_3_ axis (Fig. 1); the anisotropy patterns agree with the experiments. For 1-Sm, CASSCF-SO calculations give *g*_*x*,*y*,*z*_ = 0.4, 0.4, 0.6 for the lowest Kramers doublet. This has the opposite sense of anisotropy (*g*_*x*,*y*_ < *g*_*z*_) to the experimental data (*g*_*x*,*y*_ > *g*_*z*_), but given the numerically similar and very low (all < 1) effective *g*-values, such a switch in anisotropy will be very sensitive to subtle state mixing effects. Nevertheless, given the axially symmetric form of the EPR spectrum, *g*_*z*_ must be associated with the pseudo-C_3_ axis.

### Pulsed EPR spectroscopy

**Fig. 5:**
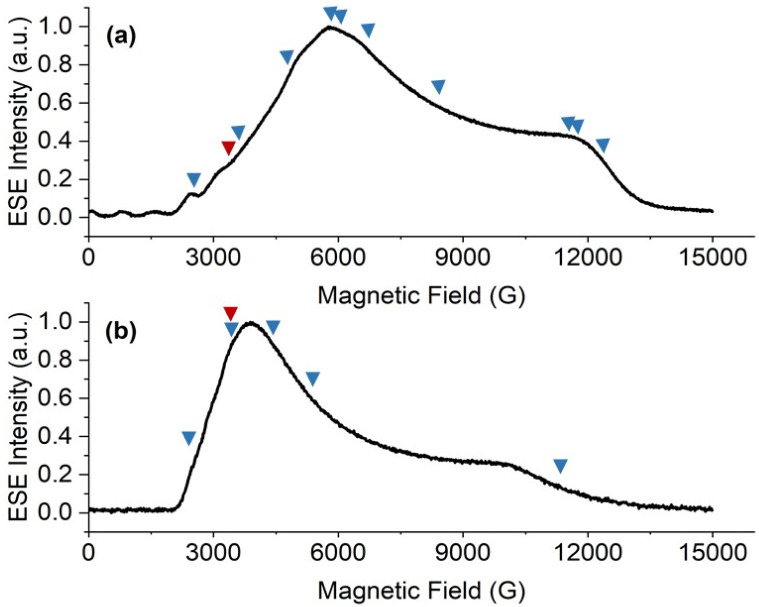
X-band (9.7 GHz) EDFS spectra of (a) 1-Nd and (b) 1-Ce, measured at 5 K from toluene : hexane (9 : 1) solution. Arrows indicate the observer positions at which *T*_1_ (blue), *T*_m_ (blue) and Hyscore (red) were measured.

Echo-detected field-swept spectra (EDFS) of 1-Nd, 1-Ce and 1-Sm (in toluene-hexane) are observed up to 10 K (see ESI† for all pulsed EPR spectra). These were recorded by integration of the Hahn echo generated with the standard pulse sequence π/2–*τ*–π–echo, where π/2 and π are microwave pulses and *τ* is the inter-pulse delay.^19^ The EDFS spectra at 5 K for 1-Nd, 1-Ce and 1-Sm (Fig. 5, S28 and S29†) are consistent with the CW spectra, Q-band EDFS are consistent with the rhombic *g*-values of 1-Nd and 1-Ce (Fig. S30†).

Measurements of the spin-lattice (*T*_1_) and phase-memory (*T*_m_) relaxation times were performed at X-band on 10 mM frozen solution samples of 1-Nd, 1-Ce and 1-Sm at 5 K. *T*_1_ data were recorded with an inversion recovery pulse sequence, and the data fitted with a bi-exponential function (see ESI eqn (3);† Fig. S34 and S35†), where the fastest process is assigned to the spectral diffusion and the slow process is attributed to the spin-lattice relaxation.^36^ The *T*_1_ value for 1-Nd is field-dependent and reaches a maximum value of 12 μs at the highest field *g*-value (12 360 G, Table S3†). Measured at the maximum of the EDFS (6040 G), *T*_1_ is 5 μs. Much longer maximum *T*_1_ times of 89 and 150 μs were obtained for 1-Ce (at the high-field *g*-value, 11 228 G) and 1-Sm (at the low-field *g*-value, 6936 G), respectively (Tables S4 and S5†). For comparison, *T*_1_ measured at the EDFS maxima are 78 and 118 μs for 1-Ce and 1-Sm, respectively. We note that the measured *T*_1_ data are anisotropic (Fig. S34 and S35, Tables S3–S5†), and the anisotropy (evaluated as the ratio of *T*_1,‖_/*T*_1,⊥_)^37^ is most significant for 1-Nd which also has the greatest *g*-anisotropy.

*T*_m_ was determined by fitting Hahn echo decays to a stretched exponential function (see ESI eqn (1); Fig. S31–S33†).^38^ The maximum *T*_m_ relaxation times observed are 0.7, 1.0 and 1.7 μs for 1-Nd, 1-Ce and 1-Sm, respectively (Tables S3–S5†), in each case measured at the highest field *g*-value. *T*_m_ measured at the EDFS maxima are 0.5, 0.6 and 1.6 μs, respectively.

An interesting comparison can be made between 1-Nd and its valence isoelectronic An^3+^ analog 1-U. Complex 1-U has *T*_1_ and *T*_m_ times of 860 and 0.8 μs, measured at the EDFS maximum under similar conditions,^21^ compared to 5 and 0.5 μs for 1-Nd. Hence, *T*_1_ for the Ln^3+^ 4f^3^ complex is more than two orders of magnitude *shorter* than for its An^3+^ 5f^3^ analogue. In general, electron spin-lattice relaxation times are expected to decrease going down the Periodic table, as modulation of the electronic structure by vibrational modes (which is a dominant factor for *T*_1_ relaxation in immobilised samples^24^) impacts the orbital angular momentum. This is connected to the spin *via* spin–orbit coupling (SOC), and SOC increases with atomic number;^39^ the values for for Nd^3+^ and U^3+^ are 900 and *ca*. 1700 cm^−1^, respectively.^40^ It seems reasonable to assume that the slower *T*_1_ relaxation of 1-U is due to the partial quenching of the orbital angular momentum by the larger crystal field interaction for U^3+^*cf*. Nd^3+^. This is promising for future pulsed EPR studies of An-containing materials. The 4f^1^ complex 1-Ce also has a much shorter *T*_1_ than its An^3+^ analogue 1-Th (*T*_1_*ca*. 21 ms under similar conditions):^21^ this is due to the orbital singlet 6d^1^ configuration of 1-Th,^26^ compared to 4f^1^ for 1-Ce.

Comparing the relaxation data for the 1-Ln series, it could be inferred that 1-Nd has the shortest *T*_1_ because it has the largest ground state orbital angular momentum (*L* = 6) and indeed the largest total angular momentum (*J* = 9/2). However, comparison with *T*_1_ data for these Ln^3+^ ions at similar concentrations and temperatures in water/ethanol glasses gives a different trend, with Nd^3+^ having the longest *T*_1_.^41^ This emphasises the importance of the crystal field on the relaxation behaviour of Ln^3+^ ions. In our case, of the three complexes studied, 1-Nd has, by some margin, the lowest energy excited state and indeed the smallest energy spread of *m*_J_ states desite having the largest multiplicity (Tables S10–S13†). More densely packed electronic states renders the states more sensitive to perturbations (following textbook perturbation theory arguments), and so we can speculate that this exposes 1-Nd to more influences from spin-vibration coupling, and thus causes a shorter *T*_1_.

The *T*_m_ values for 1-Ln complexes studied are all similar, around the 1 μs mark. These are ample to allow further investigation of the complexes by multi-pulse microwave sequences. In order to quantify the weak hyperfine interactions between the electron spin(s) and surrounding ^1^H and ^13^C nuclei, we employed two-dimensional hyperfine sub-level correlation (HYSCORE) spectroscopy, which uses a four-pulse spin echo envelope modulation sequence; π/2–*τ*–π/2–*t*_1_–π–*t*_2_–π/2–echo, with *t*_1_ and *t*_2_ independently varied.^42^ In the HYSCORE experiment the first two π/2 pulses generate nuclear coherences, which are then transferred between electron spin states by the π pulse. In the 2D frequency domain spectrum, cross-peaks appear for weakly-coupled nuclei (2|*ν*_*n*_| > |*A*|) in the (+, +) quadrant straddling the nuclear Larmor frequencies (*ν*_*n*_). Ridges in the spectra are due to the anisotropic hyperfine couplings.^43^

**Fig. 6:**
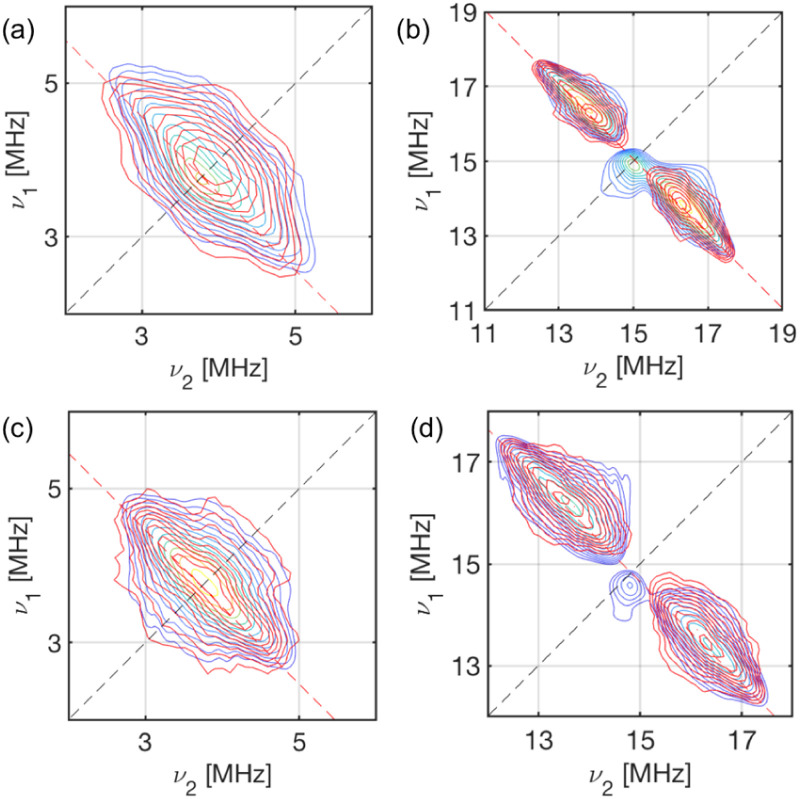
X-Band HYSCORE spectra for 1-Nd (a and b; static field *B*_0_ = 353.0 mT) and 1-Ce (c and d; *B*_0_ = 348.2 mT) at 5 K. (a and c) ^13^C region (blue) for 1-Nd and 1-Ce, respectively, and calculated spectra (red) based on a dipolar model including C1–C5 (Tables S8 and S9†); (b and d) ^1^H region (blue) for 1-Nd and 1-Ce, respectively, and calculated spectra (red) based on a dipolar model including H2, H4 and H5 (Tables S8 and S9†). The red dashed anti-diagonal lines indicate the nuclear Larmor frequency for ^13^C (a and c) or ^1^H (b and d).

X-band HYSCORE spectra measured at static magnetic fields corresponding to orientations in the molecular *xy* plane for 1-Nd and 1-Ce (*B*_0_ = 353.0 and 348.2 mT, respectively) reveal signals from ^13^C and ^1^H nuclei (Fig. 6). HYSCORE signals for 1-Sm were too weak to detect when measured under similar conditions; this does not appear to be relaxation limited given the similar *T*_m_ (see above), and may be a function of the very low effective *g*-values.^24^ The ^13^C features for 1-Nd and 1-Ce are similar, consisting of a ridge extending beyond the ^13^C Larmor frequency by *ca.* ± 1 MHz. The ^1^H features are also similar, with a ridge extending *ν*_*n*_ ± 2.5 MHz.

In a first attempt to model the HYSCORE data of 1-Ce and 1-Nd, we used a simple point dipolar approach assuming the electron spin density to be located at the Ln^3+^ ion, calculated (ESI, eqn (4)†) using the experimental *g*-values and the atomic coordinates from XRD (thus neglecting structural relaxation in solution). The calculated point dipolar hyperfine values for the Cp-ring hydrogen and carbon atoms (Tables S8 and S9†) were then used with EasySpin^44,45^ to calculate HYSCORE spectra. The calculated spectra show excellent agreement with the experimental data (Fig. 6).

Comparing the HYSCORE data of 4f 1-Ce and 1-Nd to their 5f analogues 1-An, ^13^C HYSCORE spectra measured for 1-Th under equivalent conditions have ridges that spread *ν*_*n*_ ± 2.4 MHz,^21^ substantially larger than for 1-Ce. We did not have sufficient signal to measure the full ^13^C hyperfine pattern for 1-U, but we did measure well-resolved ^1^H ridges in the *g*_*x*,*y*_ region:^21^ these spanned *ν*_*n*_ ± 2.5 MHz, which is much larger than for 1-Nd. The spectra for 1-Th and 1-U could not be modelled on a simple point dipole basis, requiring significant additional contributions to the hyperfine interactions.

We futher confirmed these results using CASSCF-SO calculations (see ESI†). To compare with the HYSCORE experiment, we approximate solution phase structures by optimising the molecular geometry of each complex in the gas-phase using density-functional theory (DFT) methods (see ESI† for details), and then use the optimised geometries to perform another set of minimal CASSCF-SO calculations; note that these calculations explicitly include CF, SO and interelectronic repulsion effects of the f-shell ground configuration. We then use the Hyperion package to calculate the relativistic hyperfine coupling tensors directly for the nuclei of interest (Tables S14 and S15†);^29^ note that the Hyperion method implicitly includes all through space dipolar and Fermi contact terms, as well as relativistic paramagnetic spin–orbit terms. Inspection of the computed hyperfine values show the C(Cp) and H(Cp) atoms couple more strongly than any other Cp^tt^ ligand atoms. Hence, we simulate ^13^C and ^1^H HYSCORE spectra using five C and three H nuclei from one Cp^tt^ ligand, again using EasySpin; these calculations provide excellent reproductions of the experimental data (Fig. S36†). The calculated Mulliken spin populations (Table S16†), show that only ∼0.1% of the total spin density is transferred to the C1–C5 atoms of the three Cp^tt^ ligands. However, we note that the density does increase slightly with increasing Ln^3+^ atomic number (1-Ce < 1-Nd < 1-Sm).

## Conclusions

We have synthesised a family of Ln^3+^ complexes [Ln(Cp^tt^)_3_] (1-Ln; Ln = La, Ce, Nd, Sm) using a salt metathesis route. The solid-state structures of 1-Ln reveal that the distances between the Ln^3+^ centres and the Cp^tt^ centroids decrease regularly from La^3+^ to Sm^3+^ due to the ionic radii of Ln^3+^ ions decreasing across the series. Complexes 1-La and 1-Nd exhibit *pseudo*-trigonal planar geometries, which is consistent with the previously reported structures of 1-Ce,^27^1-Sm ^28^ and 1-Yb.^31^ Continuous wave and pulsed EPR studies were performed on 1-Nd, 1-Ce, and 1-Sm. The CW spectra show rhombic systems with anisotropic *g*-values for both frozen solution and powder samples for 1-Nd and 1-Ce. HYSCORE spectroscopy shows resonances for ^13^C and ^1^H regions for both 1-Nd and 1-Ce, and point-dipole simulations provide excellent agreement with experimental data. CASSCF-SO calculations effectively reproduce magnetic data, and fully-*ab initio* simulations of the HYSCORE spectra are also in excellent agreement with experiment, confirming minimal spin density at ligand nuclei. The larger ligand hyperfine interactions observed for 1-Th (ref. 21) *cf*. 1-Ce are consistent with the 6d^1^*vs.* 4f^1^ configurations of Th^3+^ and Ce^3+^, respectively, and the significant radial extent of the 6d orbital(s). Comparing the results for 1-Nd with 1-U shows larger ligand hyperfines observed for the latter, highlighting the difference between 4f and 5f valence orbitals.

The results show that the interactions between Ln^3+^ ions and these cyclopentadienyl ligands in these early Ln organometallic complexes 1-Ln can be considered as being largely ionic in the sense of minimal transfer of spin density from the 4f to ligand orbitals. The results are consistent with early CW EPR studies of U^3+^ and Nd^3+^ doped fluorite^46^ (and later studies on other inorganic lattices),^47^ where superhyperfine coupling to ^19^F was observed for the former but not the latter. These are mineral lattices with hard ligands, and we have now shown related effects in molecular species with soft arene ligand sets. The results on 1-Ln also contrast with HYSCORE studies of the closely related [Yb(Cp)_3_] complex,^25^ a late Ln complex (4f^13^), which showed far more significant transfer of spin density from 4f to arene and can hence be described as being “more covalent” in this sense, despite the smaller ionic radius of the Yb^3+^ ion. This is consistent with Denning's conclusion^25^ that this “increased covalency” is due to the low reduction potential of Yb^3+^ combined with an electron-rich ligand leading to greater metal-to-ligand charge transfer character in the ground state. This highlights the importance of ligand substituents and Ln ion charge density in controlling the magnitude of 4f metal–ligand interactions, and shows that pulsed EPR spectroscopy is a sensitive probe to study these effects.

## Experimental

### Materials and methods

#### General comments

All manipulations were carried out using standard Schlenk line and glove box techniques under dry argon. Solvents were passed through columns containing alumina or were dried by refluxing over K, and were stored over K mirrors or 4 Å molecular sieves (THF) and degassed before use. For NMR spectroscopy C_6_D_6_ was dried by refluxing over K, degassed by three freeze–pump–thaw cycles, and vacuum-transferred before use. Anhydrous LnCl_3_ were purchased from Alfa Aesar and were used as received. KCp^tt^ was synthesised by literature methods,^48^ whilst 1-Ln were prepared by modification of literature procedures.^27,28^ General synthetic procedures for 1-Ln are given below; full details can be found in the ESI.†^1^H (400 and 500 MHz) and ^13^C{^1^H} (100 and 125 MHz) NMR spectra were obtained on Avance III 400 or 500 MHz spectrometers at 298 K. ^1^H NMR spectra were measured from 0 to +10 ppm for diamagnetic 1-La and from −200 to +200 ppm for paramagnetic 1-Ce, 1-Nd and 1-Sm. ATR-IR spectra were recorded as microcrystalline powders using a Bruker Tensor 27 spectrometer. Elemental analyses were performed by Mrs Anne Davies and Mr Martin Jennings at The University of Manchester School of Chemistry Microanalysis Service, Manchester, UK. UV/Vis/NIR spectroscopy was performed on samples in Youngs tap-appended 10 mm path length quartz cuvettes on an Agilent Technologies Cary Series UV/Vis/NIR spectrophotometer at 175–3300 nm. CW EPR measurements were carried out on a Bruker EMX300 spectrometer; pulsed EPR X-band studies were performed on a Bruker ElexSys E580 spectrometer. The primary Hahn-echo sequence (π/2–*τ*–π–*τ*–echo) was used for the two-pulse electron spin echo measurements, with initial π/2 and π pulse of 16 and 32 ns, respectively. For the relaxation time measurements, *T*_m_ studies were made by incrementing the *τ* time in the Hahn-echo sequence (longer pulses were used to suppress the ^1^H modulation), *T*_1_ was measured by the inversion recovery sequence (π–*t*–π/2–*τ*–π–*τ*-echo) π/2 and π pulse of 16 and 32 ns, respectively, with a fixed *τ* = 300 ns. HYSCORE measurements were performed using the four-pulse sequence (π/2–*τ*–π/2–*t*_1_–π–*t*_2_–π/2–echo), π/2 and π pulse of 16 and 32 ns, respectively, initial times *t*_1,2_ = 0.1 μs and *τ* values of 136 and 200 ns.

##### General synthesis of 1-Ln

THF (30 mL) was added to a pre-cooled (−78 °C) ampoule containing LnCl_3_ (2 mmol) and KCp^tt^ (6 mmol). The reaction mixture was allowed to reflux for 16 hours. The solvent was removed *in vacuo* and toluene (30 mL) was added. The reaction mixture was allowed to reflux for 40 hours. The resultant suspension was allowed to settle for 3 hours and filtered. The solution was concentrated to *ca*. 2 mL and stored at 8 °C to afford crystals of 1-Ln.

1-La: colourless crystals (0.550 g, 41%). Anal calcd (%) for C_39_H_63_La: C, 69.81; H, 9.47. Found (%): C, 67.03; H, 9.50. ^1^H NMR (C_6_D_6_, 400 MHz, 298 K): *δ* = 1.35 (s, 54H, C(C*H*_3_)_3_), 6.21 (s, 3H, Cp-*H*), 6.28 (s, 6H, Cp-*H*) ppm. ^13^C{^1^H} NMR (C_6_D_6_, 100 MHz, 298 K): *δ* = 32.77 (C(*C*H_3_)_3_), 33.75 (*C*(CH_3_)_3_), 110.57 (*C*H-Cp ring), 110.69 (*C*H-Cp ring), 143.45 (*C*-Cp ring) ppm. FTIR (ATR, microcrystalline): υ̃ = 2960 (s), 2899 (w), 2862 (w), 1459 (m), 1388 (w), 1356 (m), 1252 (s), 1198 (w), 1163 (w), 1088 (br, s), 1018 (s), 927 (w), 803 (s), 736 (s), 660 (w), 605 (w) cm^−1^.

1-Ce: blue crystals (0.726 g, 54%). Anal calcd (%) for C_39_H_63_Ce: C, 69.68; H, 9.45. Found (%): C, 67.49; H, 9.43. ^1^H NMR (C_6_D_6_, 500 MHz, 298 K): *δ* = −5.01 (s, 54H, C(C*H*_3_)_3_), 17.14 (s, 6H, Cp-*H*), 26.30 (s, 3H, Cp-*H*) ppm. The paramagnetism of 1-Ce precluded assignment of its ^13^C{^1^H} NMR spectrum. FTIR (ATR, microcrystalline): υ̃ = 2951 (br, s), 2899 (w), 2863 (w), 1459 (s), 1388 (m), 1356 (s), 1298 (w), 1251 (s), 1198 (m), 1164 (s), 1051 (m), 1021 (m), 927 (s), 806 (s), 738 (s), 674 (s), 659 (s), 604 (w), 556 (br, w), 480 (w), 422 (m) cm^−1^.

1-Nd: green crystals (0.460 g, 34%). Anal calcd (%) for C_39_H_63_Nd: C, 69.26; H, 9.40. Found (%): C, 65.81; H, 9.30. ^1^H NMR (C_6_D_6_, 400 MHz, 298 K): *δ* = −9.06 (s, 54H, C(C*H*_3_)_3_), 12.68 (s, 6H, Cp-*H*), 34.47 (s, 3H, Cp-*H*). The paramagnetism of 1-Nd precluded assignment of its ^13^C{^1^H} NMR spectrum. FTIR (ATR, microcrystalline): υ̃ = 2950 (s), 2899 (w), 2863 (w), 1459 (s), 1388 (w), 1356 (s), 1251 (s), 1164 (m), 1060 (br, w), 1021 (w), 927 (s), 806 (s), 737 (s), 659 (s), 605 (w), 423 (w) cm^−1^.

1-Sm: orange crystals (0.716 g, 52%). ^1^H NMR (C_6_D_6_, 400 MHz, 298 K): *δ* = −1.58 (s, 54H, C(C*H*_3_)_3_), 18.66 (s, 6H, Cp-*H*), 21.19 (s, 3H, Cp-*H*). The paramagnetism of 1-Sm precluded assignment of its ^13^C{^1^H} NMR spectrum. FTIR (ATR, microcrystalline): υ̃ = 2952 (s), 2901 (m), 2864 (w), 1460 (s), 1390 (s), 1366 (s), 1298 (s), 1250 (s), 1199 (s), 1164 (s), 1085 (w), 1060 (w), 1022 (m), 927 (s), 807 (s), 741 (s), 700 (s), 661 (s), 606 (m), 560 (m), 519 (w), 483 (w), 426 (s) cm^−1^.

## Data availability

Additional experimental details for physical and computational data associated with this manuscript (PDF). Additional research data supporting this publication are available from Figshare at https://figshare.com/10.6084/m9.figshare.23302253.

## Author contributions

F. T., E. J. L. M. and D. P. M. provided the original concept. L. N. and A.-M. A. collected and modelled EPR data. J. L., F. O., P. J. C. and J. E-K. synthesized and characterized the complexes and collected, solved and refined the single crystal and powder XRD data. F. O. further refined the single crystal XRD data and finalized cifs. L. N. and G. K. G. collected and analyzed magnetic data. M. S. O. and L. B. performed calculations. N. F. C. supervised the calculations, D. P. M. supervised the synthesis and characterization of compounds, and F. T. and E. J. L. M. supervised EPR spectroscopy. L. N., J. L., N. F. C., D. P. M., E. J. L. M. and F. T. wrote the manuscript, with contributions from all other authors.

## Conflicts of interest

There are no conflicts to declare.

## Supplementary Material

SC-015-D3SC06175B-s001

SC-015-D3SC06175B-s002
