# Supplementary material for: Metal–carbon bonding in early lanthanide substituted cyclopentadienyl complexes probed by pulsed EPR spectroscopy
Source: Chem Sci. 2024 Jan 16;15(8):3003–10. doi: 10.1039/d3sc06175b (PMC10882510; doi:10.1039/d3sc06175b)
Supplement: SC-015-D3SC06175B-s001 [file SC-015-D3SC06175B-s001.pdf]

Electronic Supplementary Material (ESI) for Chemical Science.
This journal is © The Royal Society of Chemistry 2024

Electronic Supplementary Information

Metal-carbon bonding in early lanthanide substituted cyclopentadienyl complexes probed by pulsed EPR spectroscopy

Lydia E. Nodaraki,[†,‡,§] Jingjing Liu,[†,§] Ana-Maria Ariciu,[†,‡] Fabrizio Ortu,[†] Meagan S. Oakley,[†] Letitia Birnoschi,[†] Gemma K. Gransbury,[†] Philip J. Cobb,[†] Jack Emerson-King,[†] Nicholas Chilton,[†,*] David P. Mills,[†,*] Eric J. L. McInnes,[†,‡,*] Floriana Tuna,[†,‡,*]
[†] Department of Chemistry, University of Manchester, Oxford Road, Manchester, M13 9PL, U.K.
[‡] Photon Science Institute, University of Manchester, Oxford Road, Manchester, M13 9PL, U.K.
[§] These authors contributed equally.

Contents

1. **Synthetic Procedures for 1-Ln** **S2**
2. **NMR Spectroscopy** **S4**
3. **ATR IR Spectroscopy** **S7**
4. **Crystallography** **S10**
   - 4.1 Molecular structures of **1-La** and **1-Nd** **S12**
   - 4.2 Powder XRD patterns of **1-Ln** **S13**
5. **UV/vis/NIR Spectroscopy** **S15**
6. **Magnetic Studies** **S18**
7. **EPR Spectroscopy** **S20**
   - 7.1. Continuous wave EPR measurements **S20**
   - 7.2. Pulsed EPR measurements **S21**
     - 7.2.1 Echo-detected EPR **S21**
     - 7.2.2 Phase memory time (T_m) **S23**
     - 7.2.3 Spin-lattice relaxation time (T_1) **S24**
     - 7.2.4 HYSCORE (Hyperfine sub-level correlation) Measurements **S27**
     - 7.2.5 HYSCORE Simulations (simple dipolar model) **S28**
8. **Computational Studies** **S30**
   - 8.1 Methods **S30**
   - 8.2 Calculated Properties of **1-Nd, 1-Ce** and **1-Sm** **S30**
   - 8.3 Simulated HYSCORE spectra **S32**
9. **References** **S35**

1. Synthetic Procedures for 1-Ln

[La(Cp^{tt})$_3$] (1-La). THF (30 mL) was added to a pre-cooled (−78 °C) ampoule containing $LaCl_3$ (0.491 g, 2 mmol) and KCp^{tt} (1.298 g, 6 mmol). The reaction mixture was allowed to reflux for 16 hours. The solvent was removed *in vacuo* and toluene (30 mL) was added. The reaction mixture was allowed to reflux for 40 hours. The resultant pale yellow suspension was allowed to settle for 3 hours and filtered. The pale yellow solution was concentrated to 2 mL and stored at 8 °C to afford **1-La** as colourless crystals (0.550 g, 41%). Anal calcd (%) for $C_{39}H_{63}La$: C, 69.81; H, 9.47. Found (%): C, 67.03; H, 9.50. ^{1}H NMR (C_6D_6, 400 MHz, 298 K): δ = 1.35 (s, 54H, C(C*H*$_3$)$_3$), 6.21 (s, 3H, Cp-*H*), 6.28 (s, 6H, Cp-*H*) ppm. ^{13}C{^{1}H} NMR (C_6D_6, 100 MHz, 298 K): δ = 32.77 (C(*C*H$_3$)$_3$), 33.75 (*C*(CH$_3$)$_3$), 110.57 (*C*H-Cp ring), 110.69 (*C*H-Cp ring), 143.45 (*C*-Cp ring) ppm. FTIR (ATR, microcrystalline): $\tilde{\upsilon}$ = 2960 (s), 2899 (w), 2862 (w), 1459 (m), 1388 (w), 1356 (m), 1252 (s), 1198 (w), 1163 (w), 1088 (br, s), 1018 (s), 927 (w), 803 (s), 736 (s), 660 (w), 605 (w) cm^{-1}.

[Ce(Cp^{tt})$_3$] (1-Ce). THF (30 mL) was added to a pre-cooled (−78 °C) ampoule containing $CeCl_3$ (0.493 g, 2 mmol) and KCp^{tt} (1.298 g, 6 mmol). The reaction mixture was allowed to reflux for 16 hours. The solvent was removed *in vacuo* and toluene (30 mL) was added. The dark green reaction mixture was allowed to reflux for 40 hours. The resultant suspension was allowed to settle for 3 hours and filtered. The dark purple solution was concentrated to 2 mL and stored at 8 °C to afford **1-Ce** as blue crystals (0.721 g, 54%). Anal calcd (%) for $C_{39}H_{63}Ce$: C, 69.68; H, 9.45. Found (%): C, 67.49; H, 9.43. ^{1}H NMR (C_6D_6, 500 MHz, 298 K): δ = −5.01 (s, 54H, C(C*H*$_3$)$_3$), 17.14 (s, 6H, Cp-*H*), 26.30 (s, 3H, Cp-*H*) ppm. The paramagnetism of **1-Ce** precluded assignment of its ^{13}C{^{1}H} NMR spectrum. FTIR (ATR, microcrystalline): $\tilde{\upsilon}$ = 2951 (br, s), 2899 (w), 2863 (w), 1459 (s), 1388 (m), 1356 (s), 1298 (w), 1251 (s), 1198 (m), 1164 (s), 1051 (m), 1021 (m), 927 (s), 806 (s), 738 (s), 674 (s), 659 (s), 604 (w), 556 (br, w), 480 (w), 422 (m) cm^{-1}.

[Nd(Cp^{tt})$_3$] (1-Nd). THF (30 mL) was added to a pre-cooled (–78 °C) ampoule containing $NdCl_3$ (0.501 g, 2 mmol) and KCp^{tt} (1.298 g, 6 mmol). The light blue mixture was allowed to reflux for 16 hours. The solvent was removed in *vacuo* and toluene (30 mL) was added. The reaction mixture was allowed to reflux for 40 hours. The resultant suspension was allowed to settle for 3 hours and filtered. The green solution was concentrated to 2 mL and stored at 8 °C to give **1-Nd** as green crystals (0.460 g, 34%). Anal calcd (%) for $C_{39}H_{63}Nd$: C, 69.26; H, 9.40. Found (%): C, 65.81; H, 9.30. ^{1}H NMR (C_6D_6, 400 MHz, 298 K): δ = –9.06 (s, 54H, C(C*H*$_3$)$_3$), 12.68 (s, 6H, Cp-*H*), 34.47 (s, 3H, Cp-*H*). The paramagnetism of **1-Nd** precluded assignment of its ^{13}C{^{1}H} NMR spectrum. FTIR (ATR, microcrystalline): $\tilde{\upsilon}$ = 2950 (s), 2899 (w), 2863 (w), 1459 (s), 1388 (w), 1356 (s), 1251 (s), 1164 (m), 1060 (br, w), 1021 (w), 927 (s), 806 (s), 737 (s), 659 (s), 605 (w), 423 (w) cm^{-1}.

[Sm(Cp^{tt})$_3$] (1-Sm). THF (30 mL) was added to a pre-cooled (−78 °C) ampoule containing $SmCl_3$ (0.513 g, 2 mmol) and KCp^{tt} (1.298 g, 6 mmol). The reaction mixture was allowed to reflux for 16 hours. The bright yellow solvent was removed *in vacuo* and toluene (30 mL) was added. The reaction mixture was allowed to reflux for 40 hours. The resultant suspension was allowed to settle for 3 hours and filtered. The orange solution was concentrated to 2 mL and stored at 8 °C to afford **1-Sm** as orange crystals (0.716 g, 52%). ^{1}H NMR (C_6D_6, 400 MHz, 298 K): δ = –1.58 (s, 54H, C(C*H*$_3$)$_3$), 18.66 (s, 6H, Cp-*H*), 21.19 (s, 3H, Cp-*H*). The paramagnetism of **1-Sm** precluded assignment of its ^{13}C{^{1}H} NMR spectrum. FTIR (ATR, microcrystalline): $\tilde{\upsilon}$ = 2952 (s), 2901 (m), 2864 (w), 1460 (s), 1390 (s), 1366 (s), 1298 (s), 1250 (s), 1199 (s), 1164 (s), 1085 (w), 1060 (w), 1022 (m), 927 (s), 807 (s), 741 (s), 700 (s), 661 (s), 606 (m), 560 (m), 519 (w), 483 (w), 426 (s) cm^{-1}.

2. NMR Spectroscopy

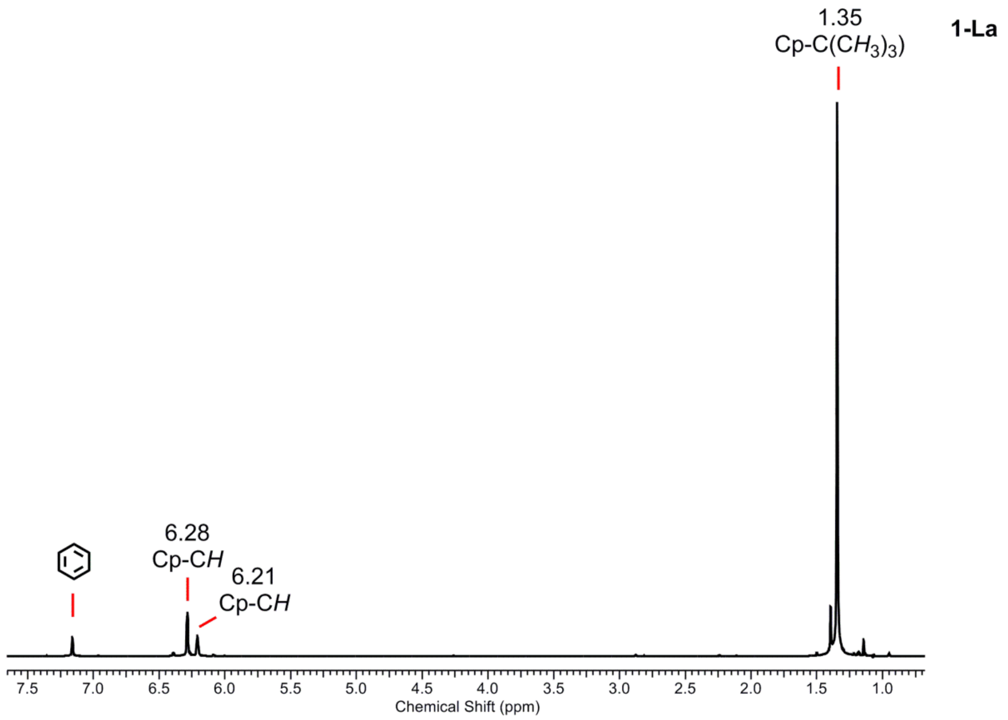

Figure S1. ^{1}H NMR spectrum of complex **1-La** in C_6D_6 zoomed in the region 1 and 7.5 ppm. Solvent residual marked.

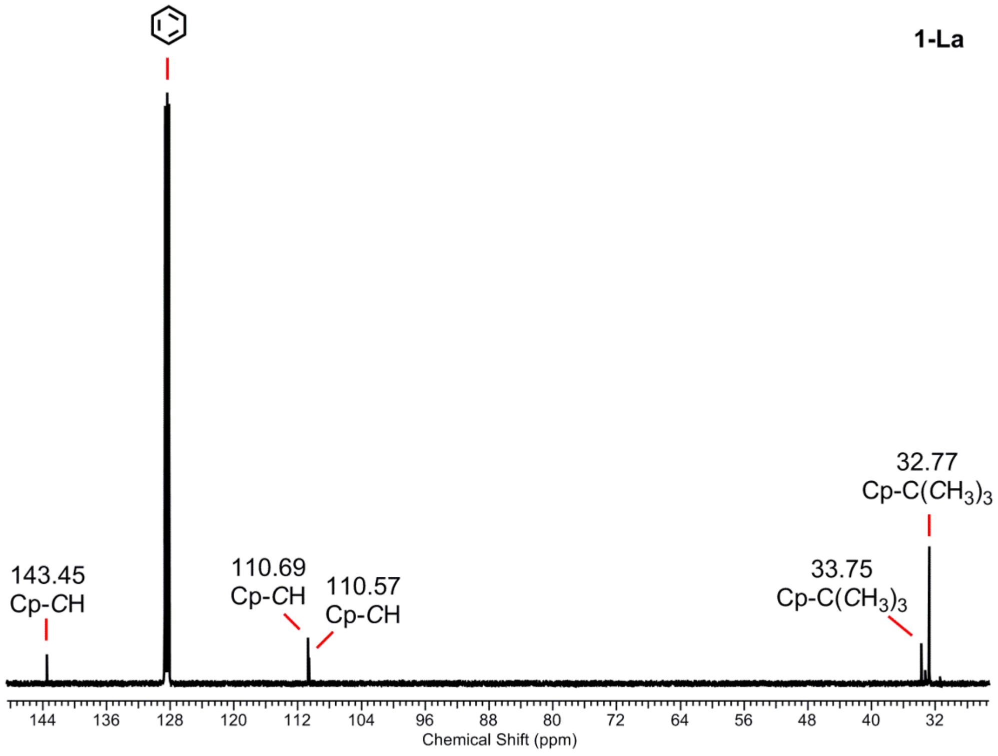

Figure S2. $^{13}C\{^{1}H\}$ NMR spectrum of complex **1-La** in C_6D_6. Solvent residual marked.

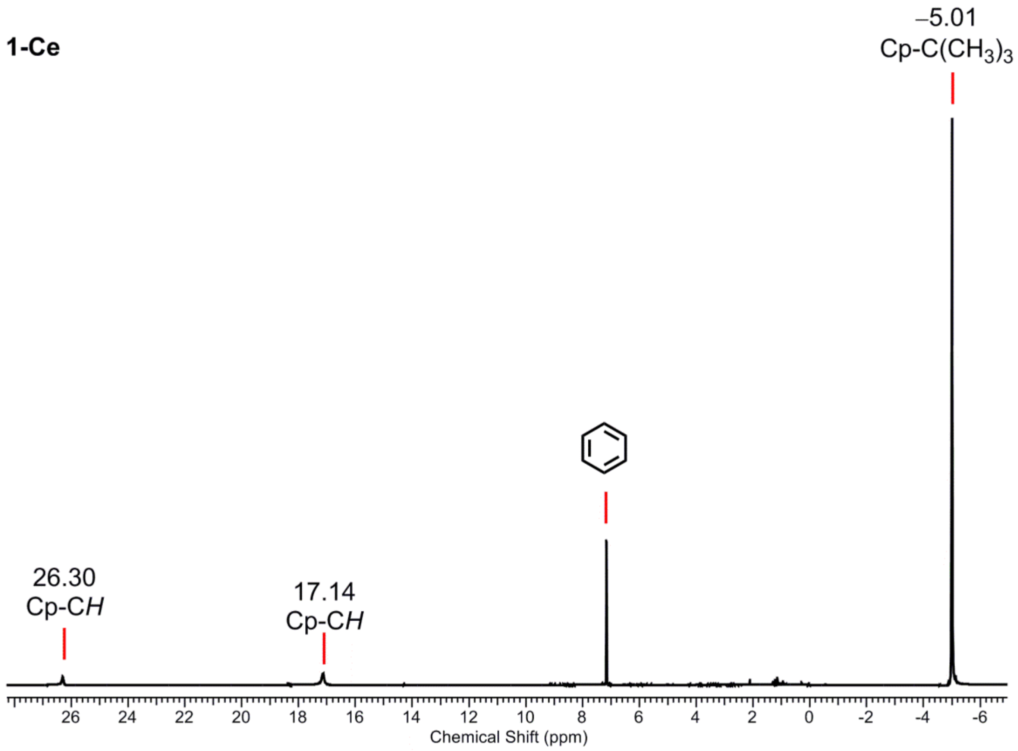

Figure S3. ^{1}H NMR spectrum of complex **1-Ce** in C_6D_6 zoomed in the region –7 and 28 ppm. Solvent residual marked; minor diamagnetic impurities can be seen between 0 and 4 ppm.

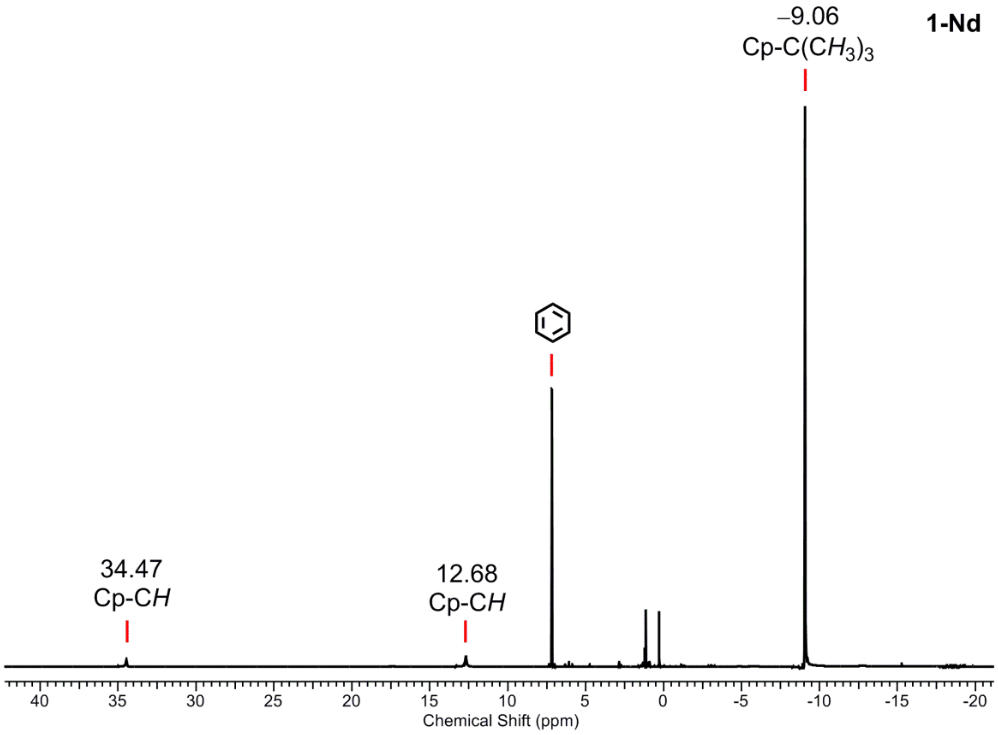

Figure S4. ^{1}H NMR spectrum of complex **1-Nd** in C_6D_6 zoomed in the region –20 and 40 ppm. Solvent residual marked; minor diamagnetic impurities can be seen between 0 and 7 ppm.

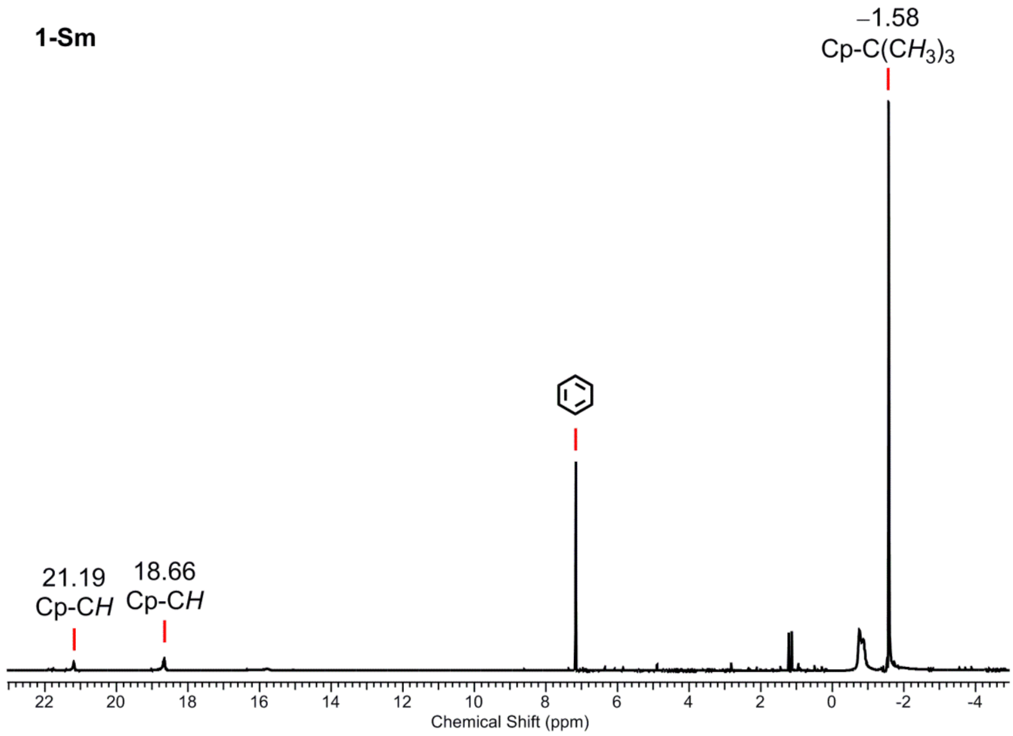

Figure S5. ^{1}H NMR spectrum of complex **1-Sm** in C_6D_6 zoomed in the region –4 and 22. Solvent residual marked; minor diamagnetic impurities can be seen between –1 and 7 ppm.

3. ATR-IR spectroscopy

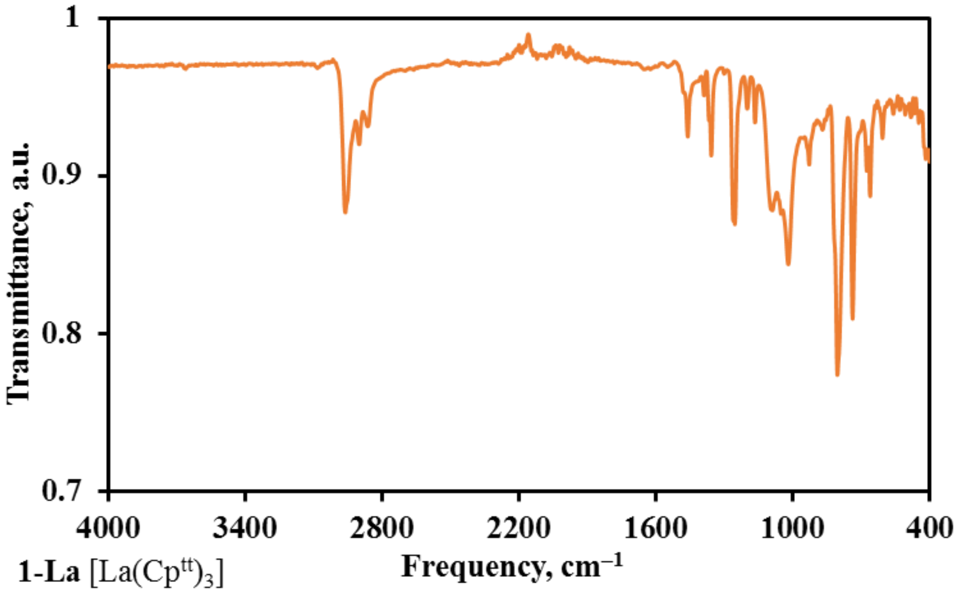

Figure S6. ATR-IR spectrum of **1-La** recorded as a microcrystalline powder.

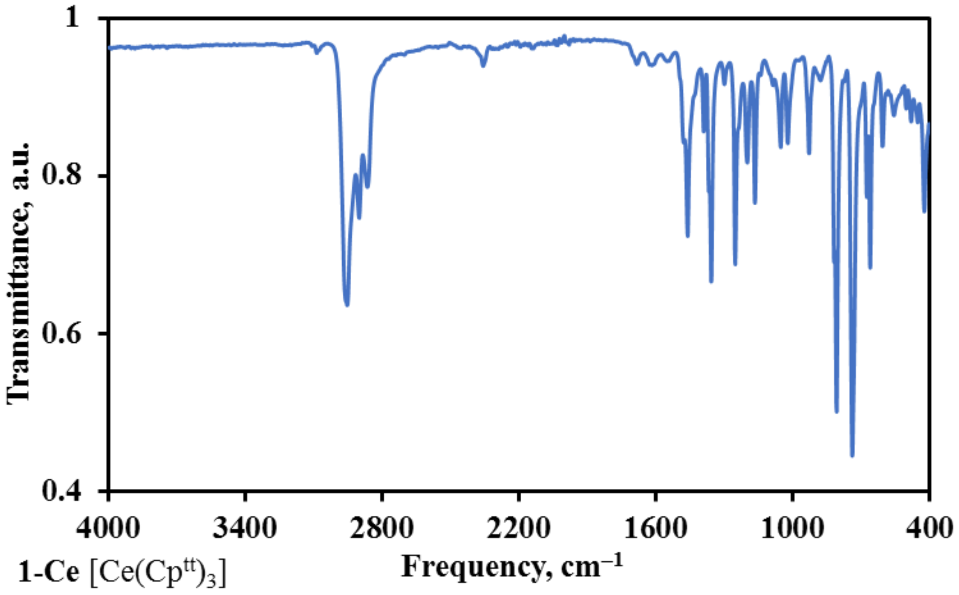

Figure S7. ATR-IR spectrum of **1-Ce** recorded as a microcrystalline powder.

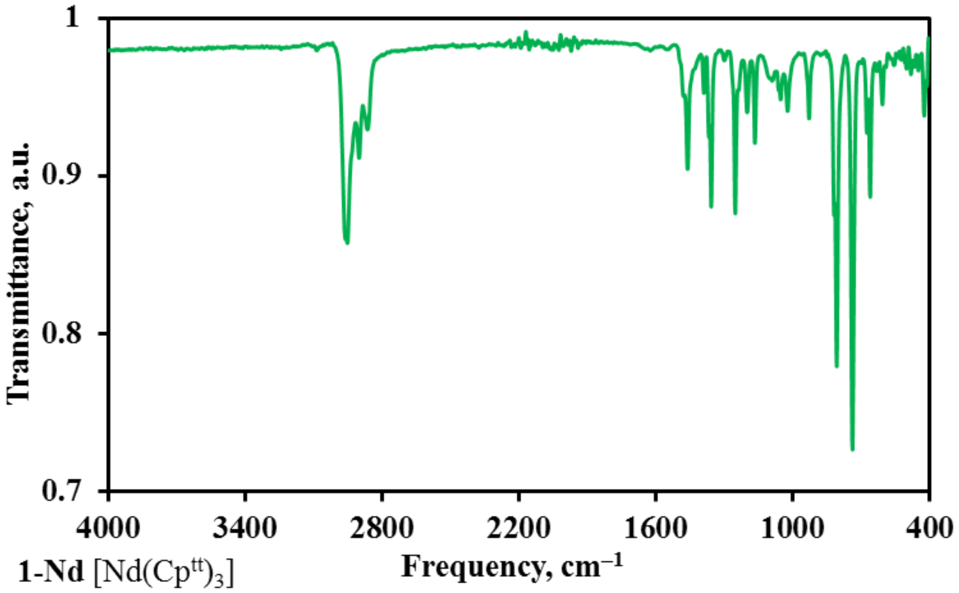

Figure S8. ATR-IR spectrum of **1-Nd** recorded as a microcrystalline powder.

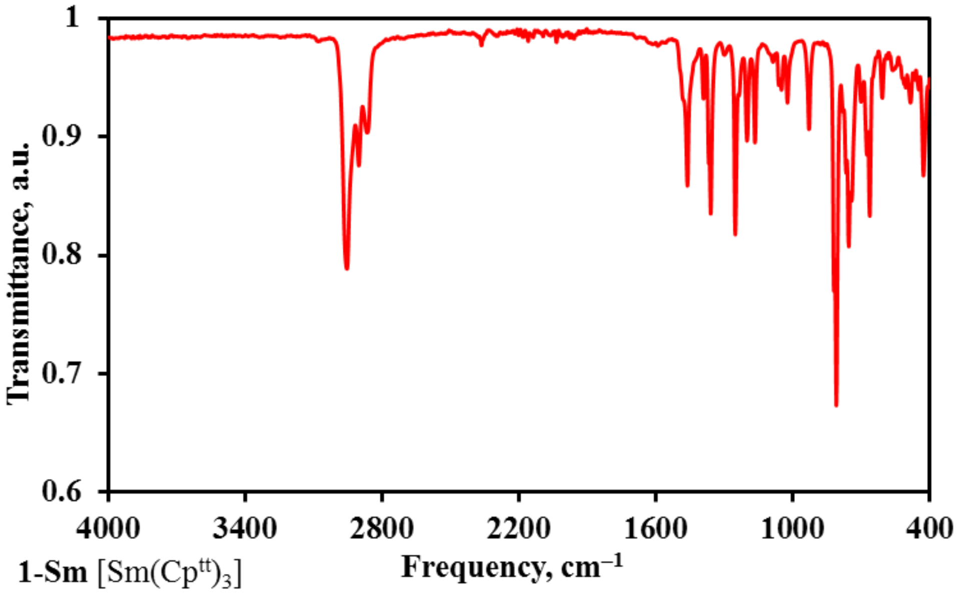

Figure S9. ATR-IR spectrum of **1-Sm** recorded as a microcrystalline powder.

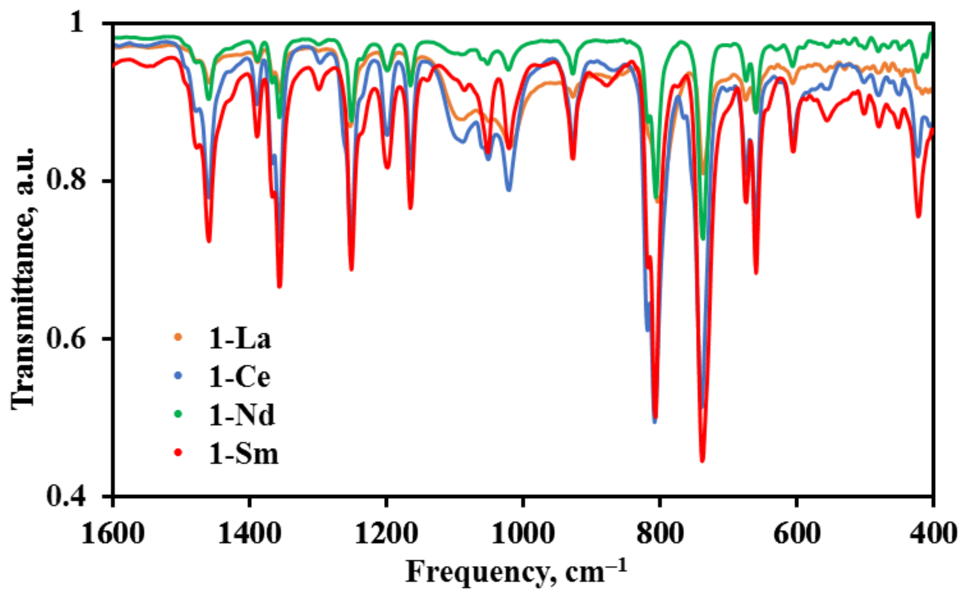

Figure S10. ATR-IR spectra of **1-La**, **1-Ce**, **1-Nd**, **1-Sm** in the region 1600–400 cm^{-1} intended to show the similarities between both spectra.

4. Crystallography

The crystal data for complexes **1-La** and **1-Nd** are compiled in **Table S1**, and are depicted in Figures S11 and S12, respectively. Crystals of **1-La** and **1-Nd** were examined using an Oxford Diffraction Supernova diffractometer with a CCD area detector and a mirror-monochromated Mo Kα radiation (λ = 0.71073 Å). Intensities were integrated from data recorded on 1° frames by ω rotation. Cell parameters were refined from the observed positions of all strong reflections in each data set. A Gaussian grid face-indexed (**1-Nd**) or multi-scan (**1-La**) absorption correction with a beam profile was applied.[1] The structures were solved using SHELXS;[2] the datasets were refined by full-matrix least-squares on all unique F^2 values,[3] with anisotropic displacement parameters for all non-hydrogen atoms, and with constrained riding hydrogen geometries; U_{iso}(H) was set at 1.2 (1.5 for methyl groups) times U_{eq} of the parent atom. The largest features in final difference syntheses were close to heavy atoms and were of no chemical significance. CrysAlisPro[1] was used for control and integration, and SHELX[2,3] was employed through OLEX2[4] for structure solution and refinement. ORTEP-3[5] and POV-Ray[6] were employed for molecular graphics. CCDC 2271676 and 2271677 contain the supplementary crystal data for this article. These data can be obtained free of charge from the Cambridge Crystallographic Data Centre via www.ccdc.cam.ac.uk/data_request/cif.

Powder XRD data of microcrystalline samples of **1-Ln** mounted with a minimum amount of fomblin were collected at 100(2) K using a Rigaku FR-X rotating anode single crystal X-ray diffractometer using Cu Kα radiation (λ = 1.5418 Å) with a Hypix-6000HE detector and an Oxford Cryosystems nitrogen flow gas system (Figures S13-S16). Data were collected between 2–70 °θ with a detector distance of 150 mm and a beam divergence of 1.5 mRad using CrysAlisPro.[1] For data processing, the instrument was calibrated using silver behenate as standard, then the data were reduced and integrated using CrysAlisPro.[1]

Table S1. Crystallographic data for **1-La** and **1-Nd**.

[a]Conventional $R = \Sigma||Fo| - |Fc||/\Sigma|Fo|$; $Rw = [\Sigma w(Fo^2 - Fc^2)^2/\Sigma w(Fo^2)^2]^{1/2}$; $S = [\Sigma w(Fo^2 - Fc^2)^2/\text{no. data} - \text{no. params})]^{1/2}$ for all data.

	**1-La**	**1-Nd**
Formula	$C_{39}H_{63}La$	$C_{39}H_{63}Nd$
Formula weight	670.80	676.13
Crystal size, mm	0.05 × 0.06 × 0.07	0.24 × 0.31 × 0.44
Crystal system	triclinic	monoclinic
Space group	*P*-1	$P2_1/n$
a, Å	10.7001(8)	10.7938(3)
b, Å	18.4935(9)	19.4422(5)
c, Å	19.7113(13)	17.8596(5)
α, °	83.299(5)	90
β, °	77.365(6)	104.394(3)
γ, °	74.640(5)	90
V, Å^3	3663.0(4)	3630.25(18)
Z	4	4
ρ_{calc}, g cm^3	1.216	1.237
μ, mm^{-1}	1.189	1.453
F(*000*)	1416	1428
No. of reflections (unique)	21453(13284)	15912 (6631)
S^a	1.00	1.06
$R_1(wR_2)$ ($F^2 > 2\sigma(F^2)$)	0.0559 (0.0926)	0.0451 (0.0968)
R_{int}	0.050	0.042
Min./max. diff map, Å^{-3}	−1.01, 1.06	−0.44, 1.74

4.1 Molecular structures of **1-La** and **1-Nd**

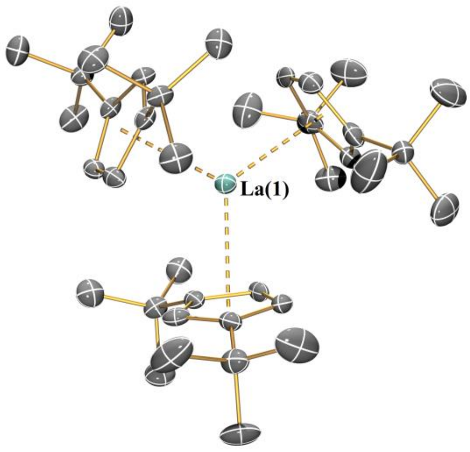

Figure S11. Molecular structure of [La(Cp^{tt})$_3$] (**1-La**) with selected labelling. Displacement ellipsoids set at 30 % probability level and hydrogen atoms are omitted for clarity. Selected distances and angles: La1···$Cp_{centroid1}$, 2.657(2) Å; La1···$Cp_{centroid2}$, 2.623(2) Å; La1···$Cp_{centroid3}$, 2.625(3) Å; $Cp_{centroid1}$···La1···$Cp_{centroid2}$, 120.38(7)°; $Cp_{centroid1}$···La1···$Cp_{centroid3}$, 118.74(8)°; $Cp_{centroid2}$···La1···$Cp_{centroid3}$, 120.88(8)°.

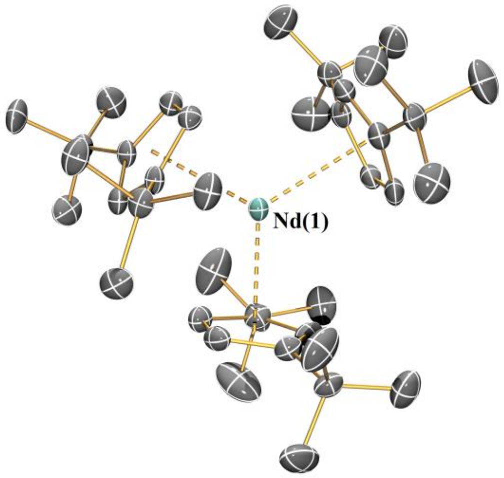

Figure S12. Molecular structure of [Nd(Cp^{tt})$_3$] (**1-Nd**) with selected labelling. Displacement ellipsoids set at 30 % probability level and hydrogen atoms are omitted for clarity. Selected distances and angles: Nd1···$Cp_{centroid1}$, 2.558(18) Å; Nd1···$Cp_{centroid2}$, 2.558(16) Å; Nd1···$Cp_{centroid3}$, 2.567(15) Å; $Cp_{centroid1}$···Nd1···$Cp_{centroid2}$, 119.68(5)°; $Cp_{centroid1}$···Nd1···$Cp_{centroid3}$, 120.60(6)°; $Cp_{centroid2}$···Nd1···$Cp_{centroid3}$, 119.71(6)°.

4.2 Powder XRD patterns of **1-Ln**

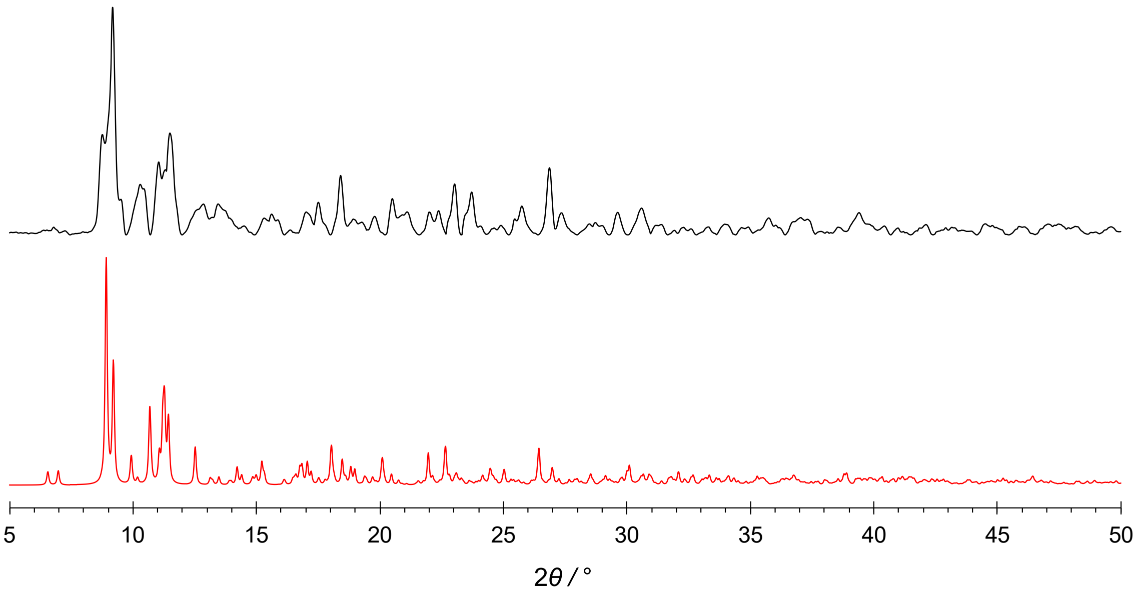

Figure S13. Simulated X-ray diffraction pattern from single crystal X-ray diffraction at 150 K (red) compared to experimental powder XRD pattern at 100 K (black) for **1-La** (arbitrary intensities).

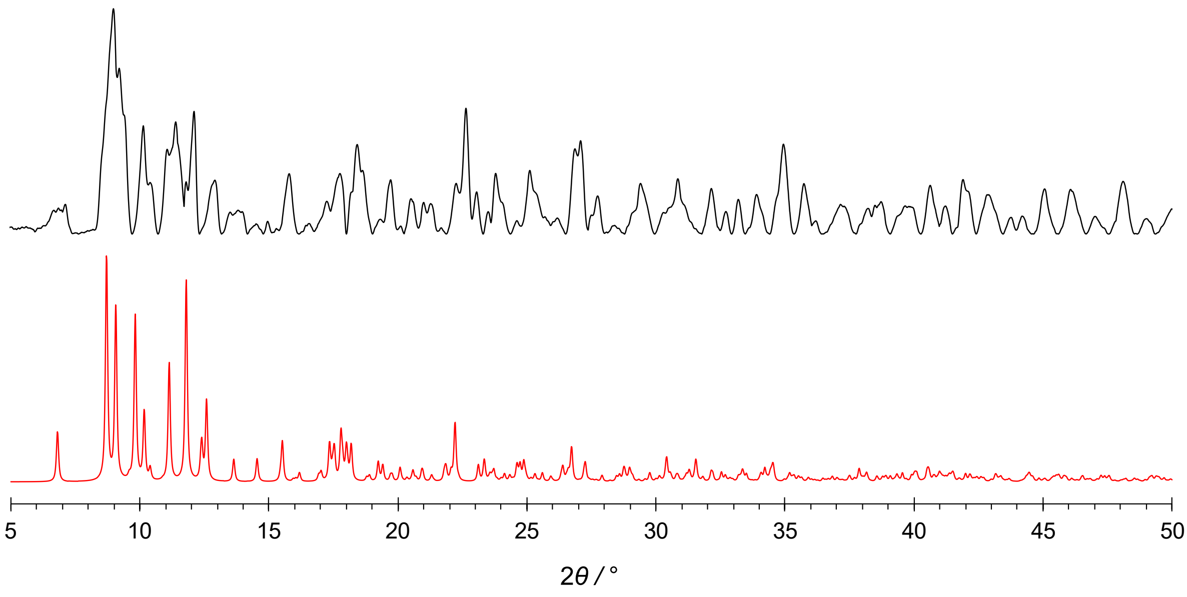

Figure S14. Simulated X-ray diffraction pattern from single crystal X-ray diffraction at 150 K (red) compared to experimental powder XRD pattern at 100 K (black) for **1-Ce** (arbitrary intensities).

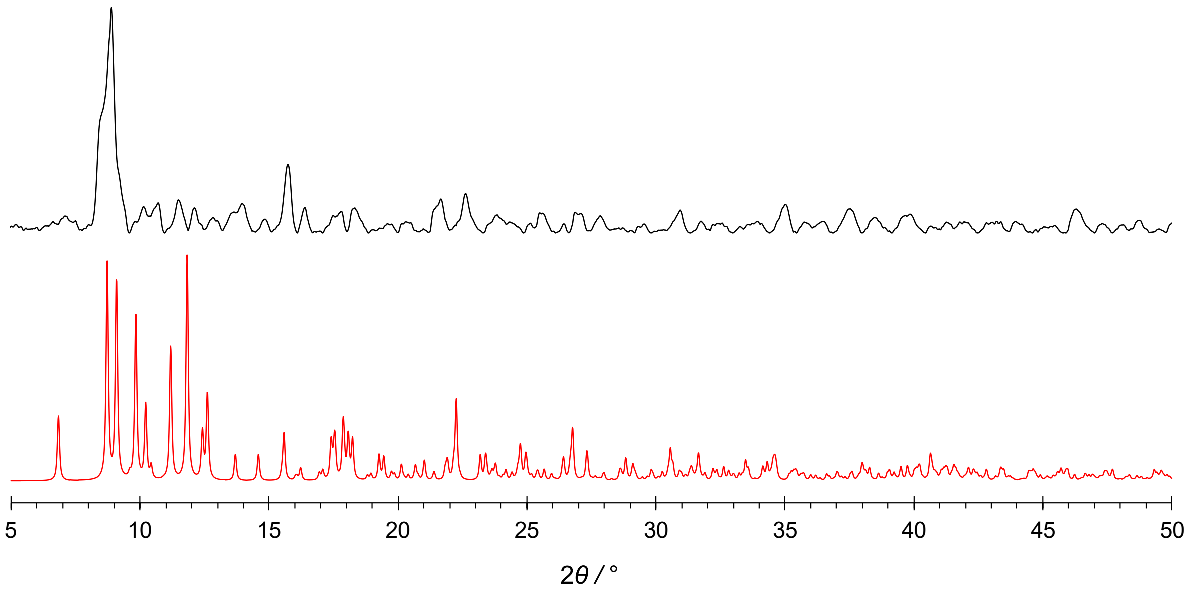

Figure S15. Simulated X-ray diffraction pattern from single crystal X-ray diffraction at 150 K (red) compared to experimental powder XRD pattern at 100 K (black) for **1-Nd** (arbitrary intensities).

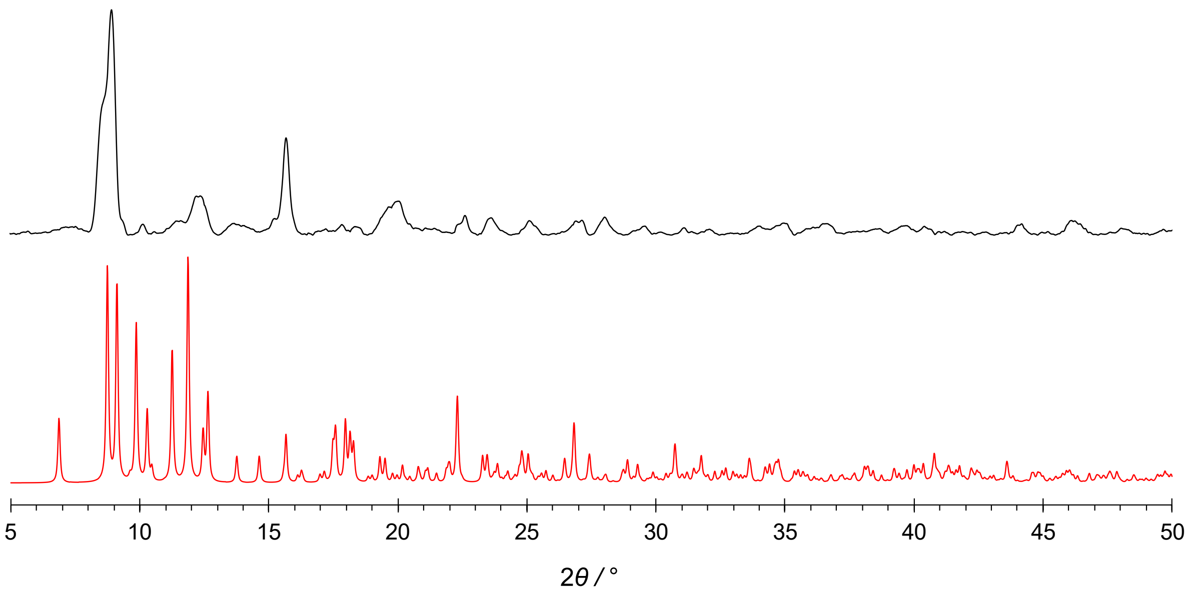

Figure S16. Simulated X-ray diffraction pattern from single crystal X-ray diffraction at 130 K (red) compared to experimental powder XRD pattern at 100 K (black) for **1-Sm** (arbitrary intensities).

5. UV/vis/NIR Spectroscopy

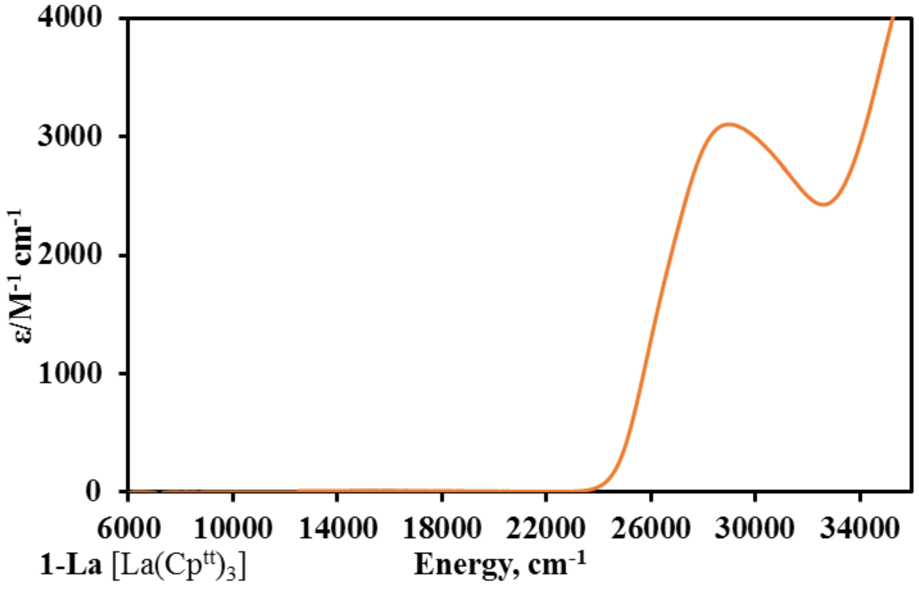

Figure S17. UV-vis-NIR spectrum of **1-La** between 36000–6000 cm^{-1} (278–1650 nm) recorded as a 0.5 mM solution in toluene.

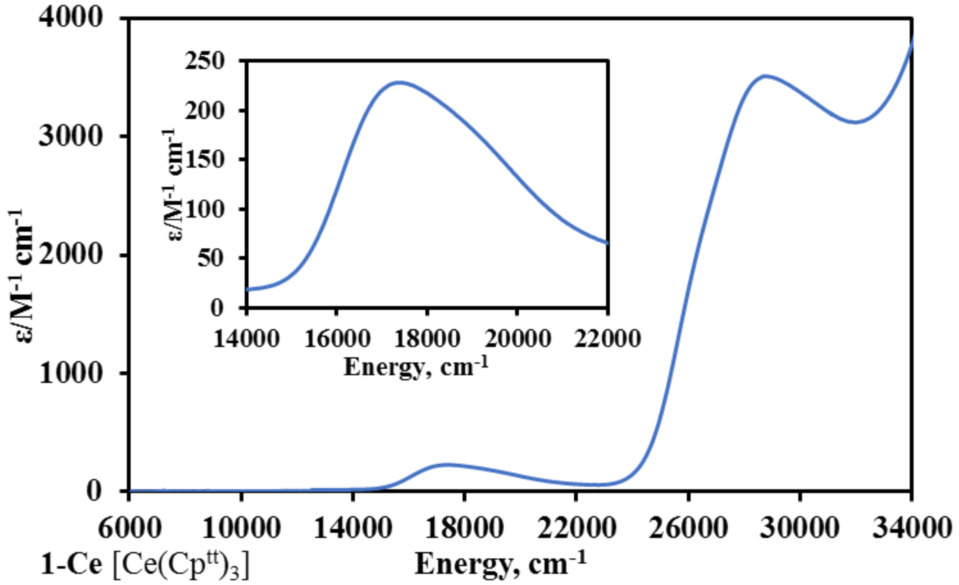

Figure S18. UV-vis-NIR spectrum of **1-Ce** between 34000–6000 cm^{-1} (295–1650 nm) recorded as a 0.5 mM solution in toluene. Inset shows the region 22000–14000 cm^{-1} (455–714 nm).

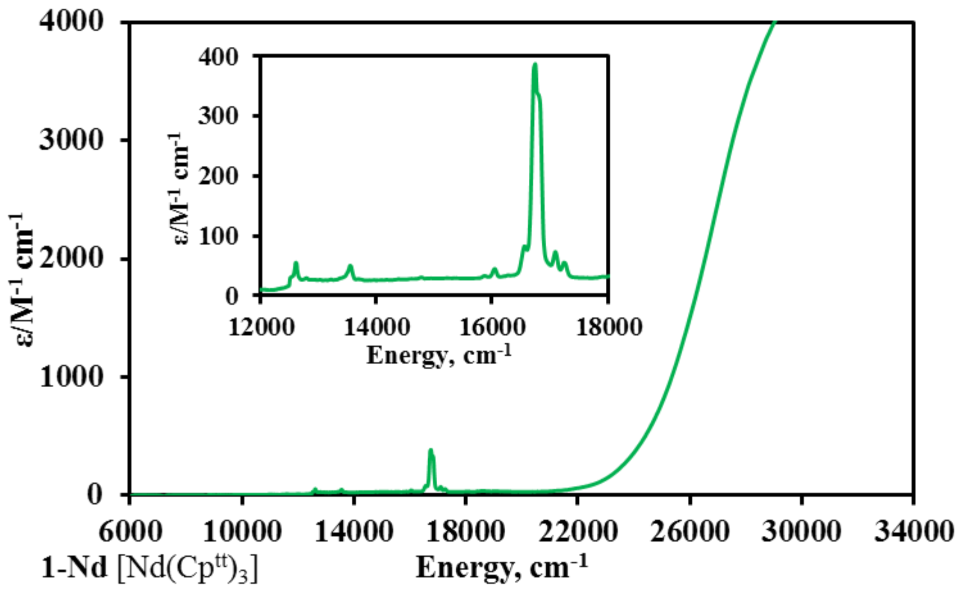

Figure S19. UV-vis-NIR spectrum of **1-Nd** between 34000–6000 cm^{-1} (295–1650 nm) recorded as a 0.5 mM solution in toluene. Inset shows the region 18000–12000 cm^{-1} (555–833 nm).

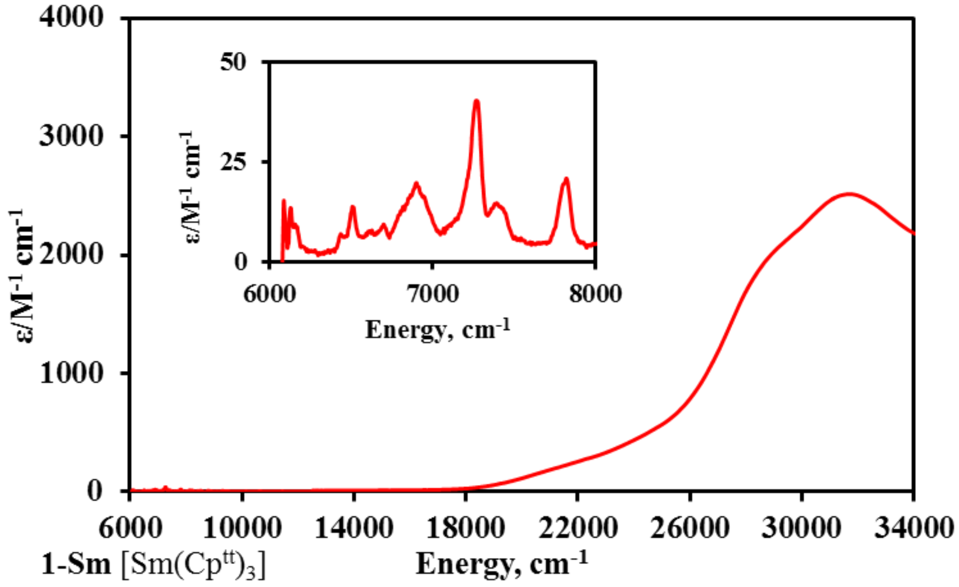

Figure S20. UV-vis-NIR spectrum of **1-Sm** between 34000–6000 cm^{-1} (295–1650 nm) recorded as a 0.5 mM solution in toluene. Inset shows the region 8000–6000 cm^{-1} (1250–1667 nm).

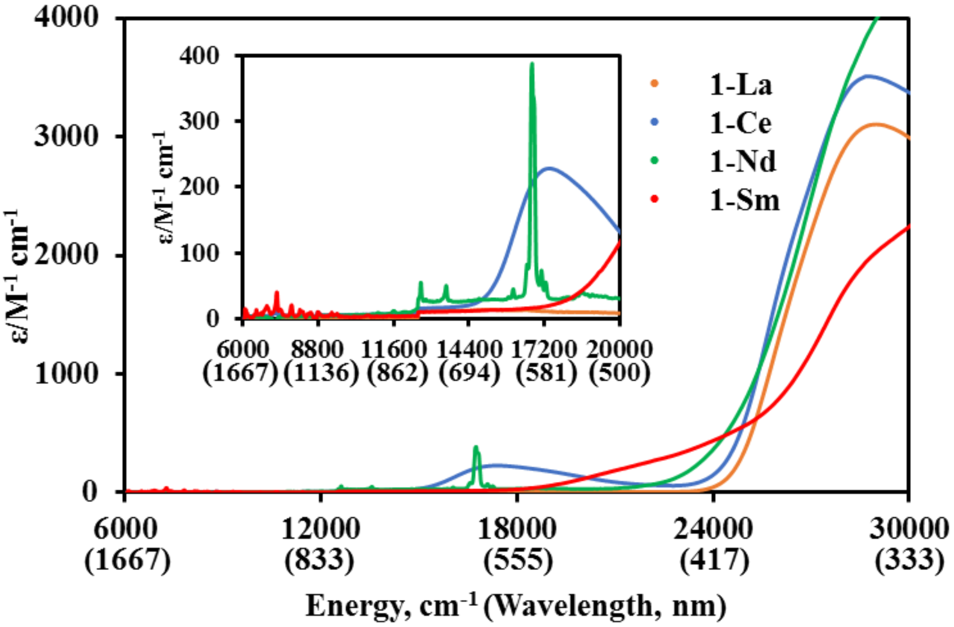

Figure S21. UV-vis-NIR spectrum of **1-La**, **1-Ce**, **1-Nd**, **1-Sm**, in the region 6000–30000 cm^{-1} (inset 6000–20000 cm^{-1}) recorded as 0.5 mM solutions in toluene at room temperature.

6. Magnetic Studies

Magnetic measurements were performed on solid-state polycrystalline samples restrained in a known amount of eicosane (to prevent orientation of the crystallites with the applied magnetic field) and flame-sealed under vacuum in a borosilicate glass NMR tube. The ampoule was mounted in the centre of a drinking straw by wrapping in Kapton tape and using friction, and the straw was fixed to the end of the sample rod. Data for **1-Nd** and **1-Ce** were collected with a Quantum Design MPMS3 SQUID magnetometer. Measurements were performed in dc scan mode with a scan length of 30 mm and scan time of 4 s. Equilibrium susceptibility measurements on **1-Ce** and **1-Nd** were performed under a dc magnetic field of 0.1 T upon cooling from 300 to 1.8 K. Complex **1-Ce** was found to slowly thermally equilibrate; for each temperature below 100 K the moment was measured continually until an equilibrium value was obtained. For compound **1-Nd**, waits were employed at each temperature to ensure equilibration: 300–100 K: 10 min, 90–5 K: 30 min; 5–2 K: 10 mins, 1.8 K: 18 min. Isothermal magnetization measurements were performed at 2 and 4 K, 0–7 T with a minimum of 10 min waits at each field point. Measurements for **1-Sm** were performed on a Quantum Design MPMS-XL7 using dc scans. Susceptibility measurements were performed between 2–300 K in a 0.5 T dc field and isothermal magnetization measurements were performed at 2 and 4 K, 0–7 T. Experimental data were corrected for the diamagnetism of the sample, using Pascal's constants, for the shape of the sample using the Quantum Design Geometry Simulator (MPMS3 data), and for the contribution of the sample holder and eicosane using calibrated blanks.

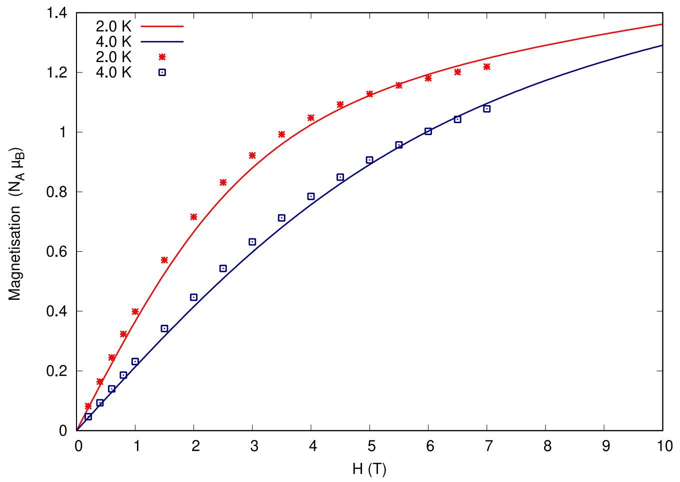

Figure S22. Field dependence of the magnetization for **1-Nd** at 2 and 4 K. Solid lines from CASSCF-SO calculations using the XRD geometry.

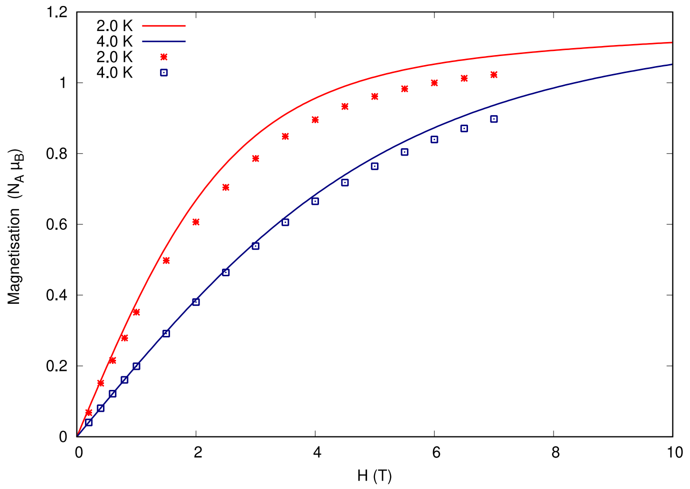

Figure S23. Field dependence of the magnetization for **1-Ce** at 2 and 4 K. Solid lines from CASSCF-SO calculations on molecule 1 using the XRD geometry (there are two independent molecules with different metrical parameters in the unit cell of **1-Ce**, so both were computed; see Section 8 for details).

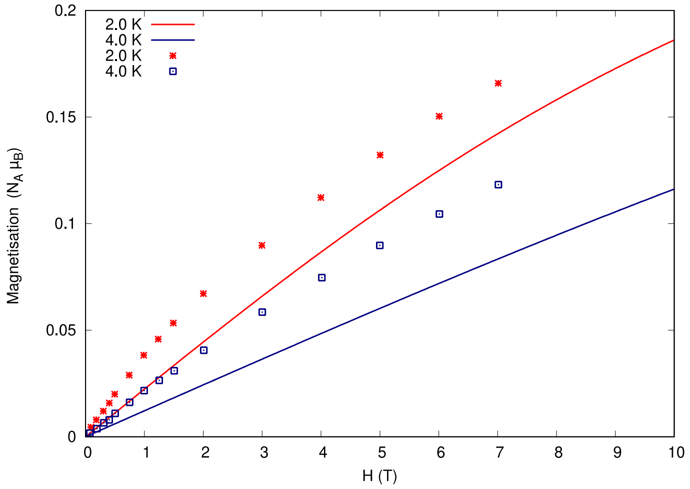

Figure S24. Field dependence of the magnetization for **1-Sm** at 2 and 4 K. Solid lines from CASSCF-SO calculations using the XRD geometry.

7. EPR Spectroscopy

7.1 Continuous-wave EPR Measurements

Continuous-wave (CW) electron paramagnetic resonance (EPR) spectra of **1-Ln** (Figs. S26-S28) were recorded on Bruker EMX 300 and Bruker ElexSys E580 EPR spectrometers operating at either X-band (*ca*. 9.4-9.8 GHz) or Q-band (*ca*. 34 GHz) mw frequencies and variable temperatures. Spectra were simulated using the EasySpin software.[7]

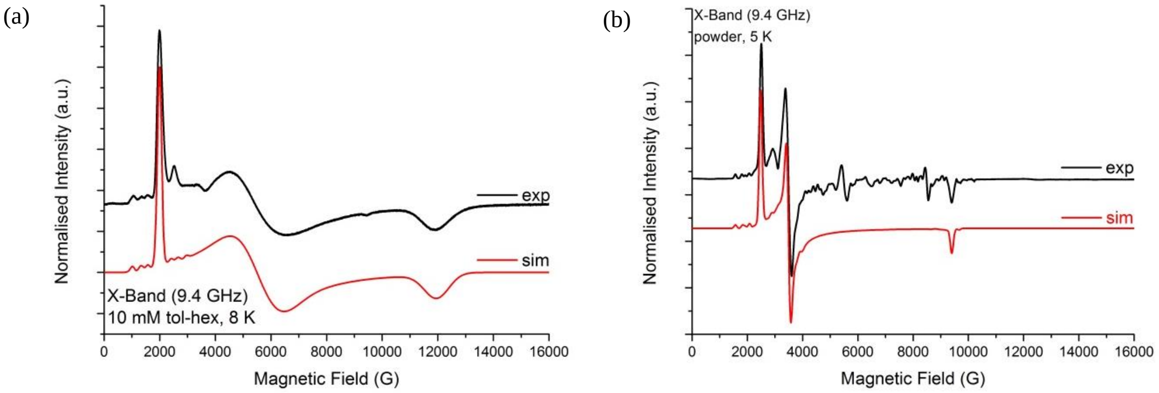

Figure S25. Experimental (black) and simulated (red) CW X-band EPR spectra of **1-Nd** (a) 10 mM toluene-hexane (9:1) solution at 8 K; (b) powder at 5 K. We observe polycrystallinity effects, previously reported for similar complexes.[8] Simulation parameters are given in Table S2.

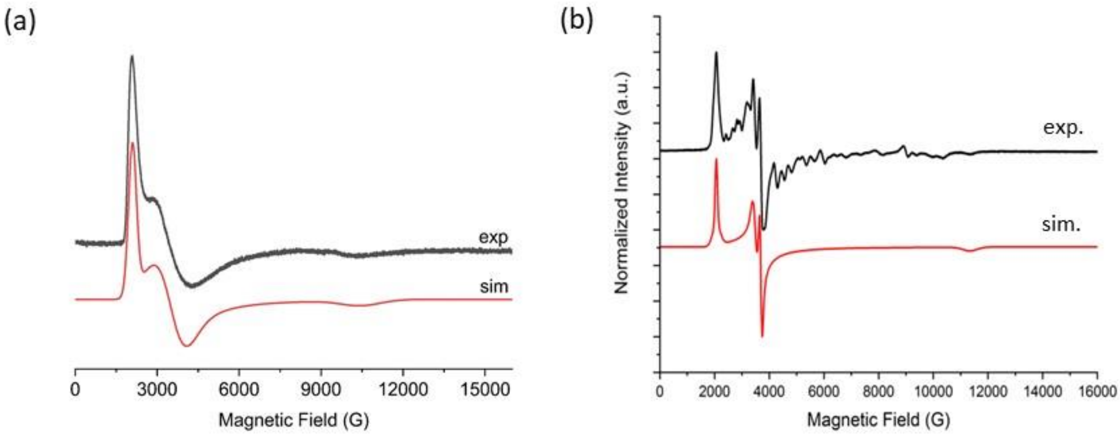

Figure S26. Experimental (black) and simulated (red) CW X-band EPR spectra of **1-Ce** (a) 20 mM toluene-hexane (9:1) solution at 10 K; (b) powder at 10 K. We observe polycrystallinity effects, previously reported for similar complexes.[8] Simulation parameters are given in Table S2.

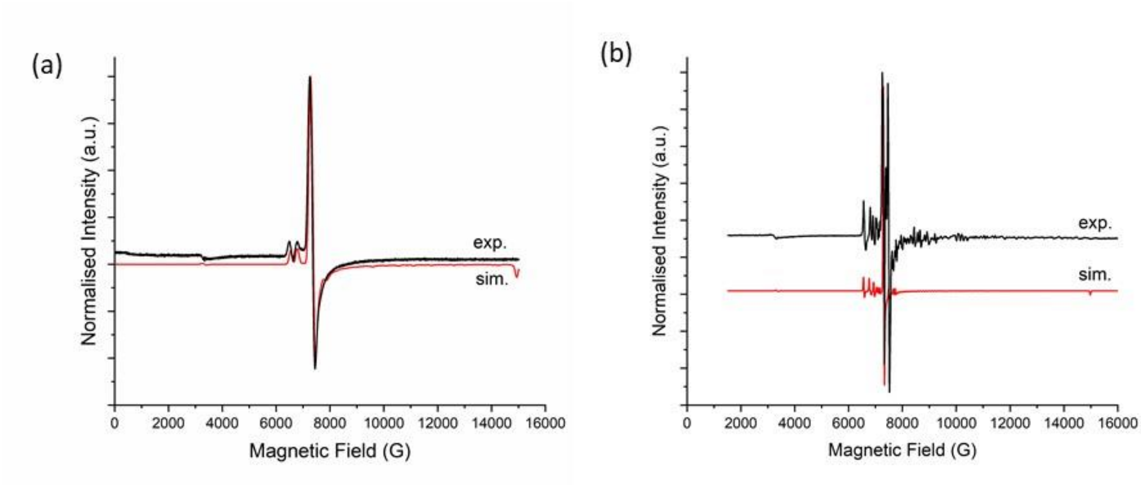

Figure S27. Experimental (black) and simulated (red) CW X-band EPR spectra of **1-Sm** (left) 20 mM toluene-hexane (9:1) solution at 20 K; (right) powder at 15 K. We observe polycrystallinity effects, previously reported for similar complexes.[8] Simulation parameters are given in Table S2.

Table S2. Hamiltonian parameters obtained from the CW simulation of **1-Ce**, **1-Nd** and **1-Sm**.

Compound	*T* (K)	*g*	|*A*| (MHz)
**1-Nd (10 mM, Tol/Hex)**	8	$g_{x/y/z}$ = 3.332/1.22/0.56	$\|A_{x/y/z}^{Nd}\|$ = 1280/700/200
**1-Nd (powder)**	5	$g_{x/y/z}$ = 2.70/1.92/0.714	$\|A_{x/y/z}^{Nd}\|$ = 980/350/100
**1-Ce (10 mM, Tol/Hex)**	10	$g_{x/y/z}$ = 3.15/1.88/0.636	-
**1-Ce (powder)**	10	$g_{x/y/z}$ = 3.24/ 1.92-1.81/ 0.588	-
**1-Sm (10 mM, Tol/Hex)**	20	$g_{x/y/z}$ = 0.917/0.917/0.477	$\|A_{x/y/z}^{Sm}\|$ = 180/180/955
**1-Sm (powder)**	15	$g_{x/y/z}$ = 0.917/0.917/0.477	$\|A_{x/y/z}^{Sm}\|$ =180/180/955

7.2 Pulsed EPR Measurements

Pulsed EPR spectra were recorded with a Bruker ElexSys E580 instrument equipped with either a MD5 or a MD4 resonator, and operating at *ca*. 9.7 GHz and various temperatures. Solution samples of different concentrations (2, 5 and 10 mM in toluene/hexane) were investigated to check reproducibility and to achieve an acceptable signal-to-noise response in HYSCORE experiments. Data were simulated with the Easy Spin software package.[7]

7.2.1. Echo-detected EPR

The echo-detected field–swept (EDFS) spectra recorded at 9.7 GHz (X-band) (Figures 5, S28 and S29) were recorded with a two-pulse primary Hahn-echo sequence ($\pi/2$ - τ - π - τ - *echo*),[9] with microwave π pulses of 32 or 64 ns, a fixed delay time τ = 300 ns, and with the variation of the static B_0 magnetic field. Those measurements at 34 GHz (Q-band; Figure S30) were recorded with microwave π pulses of 40 ns, and a fixed delay time τ = 300 ns.

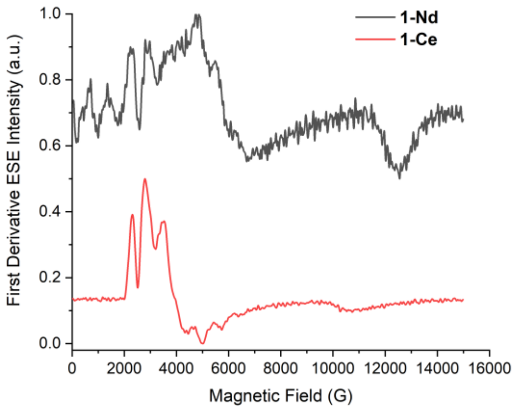

Figure S28. Calculated derivative of the X-band (9.7 GHz) EDFS spectra in Figure 5, for frozen solutions of **1-Nd** and **1-Ce** at 5 K.

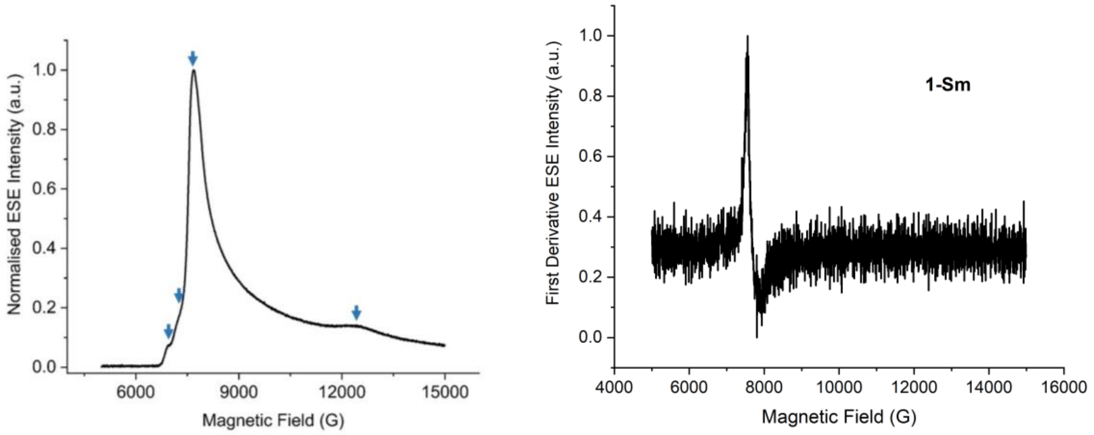

Figure S29. (left) X-band (9.7 GHz) EDFS spectrum of a frozen solution **(**10 mM in 9:1 toluene-hexane at 5 K) of **1-Sm**. Arrows indicate the observer field positions where T_1 and T_m were measured. (right) Calculated derivative for the same EDFS spectrum.

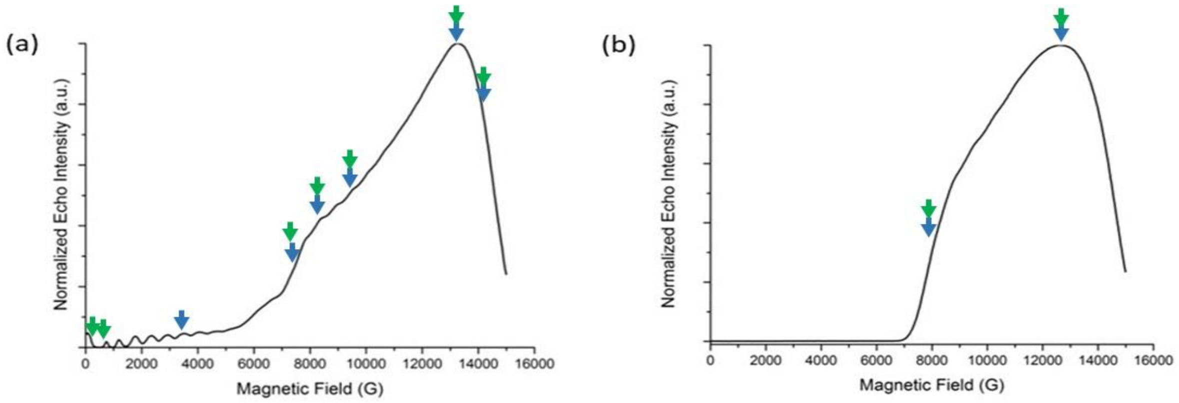

Figure S30. Q-band (34 GHz) EDFS spectra of frozen solutions **(**10 mM in 9:1 toluene-hexane) of (a) **1-Nd** at 3 K and (b) **1-Ce** at 5 K. Arrows indicate the observer positions at which T_1 (blue) and T_m (green) were measured.

7.2.2. Phase Memory Time (T_m)

Electron spin echo envelope modulation (ESEEM) measurements involved monitoring the echo intensity generated with a primary Hahn-echo sequence as a function of τ. A similar pulse sequence was used to measure the phase memory time, T_m, with the difference that longer pulse durations (up to 128 ns) were necessary to suppress possible ^{1}H nuclear modulation effects in the echo decays (Figures S31-S33). T_m was determined by least squares fitting of the experimental echo decay data using a stretched exponential function with a solver based on the Levenberg-Marquardt algorithm.

The fitting function used was:

$$Y(2\tau) = Y(0)e^{(-2\tau/T_m)X} \text{ (Equation 1)}$$

or, for strongly modulated data,

$$Y(2\tau) = Y(0)e^{(-2\tau/T_m)X}(1 + k\sin(\omega t + \Phi)) \quad \text{(Equation 2)}$$

where k is the modulation depth, ω is the Larmor angular frequency of a nucleus I coupled to the electron spin, ϕ is the phase correction, X is the stretching parameter, $Y(2\tau)$ is the echo integral for a pulse separation τ, and $Y(0)$ is the echo intensity extrapolated to $\tau = 0$.[10-13]

The extracted T_m times are given in Tables S3-S7.

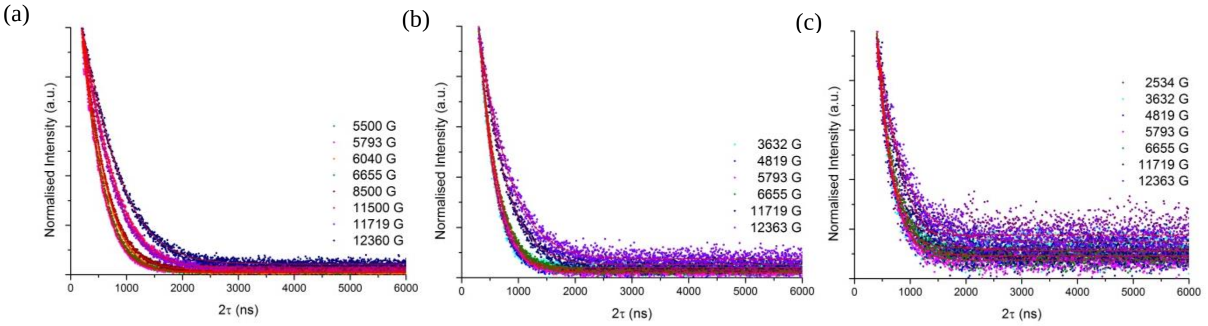

Figure S31. Normalized Hahn echo signal intensities of **1-Nd** as a function of the inter-pulse delay 2τ at different magnetic fields at 5 K, measured with π/τ (ns) of (a) 32/200; (b) 64/300, and (c) 128/400.

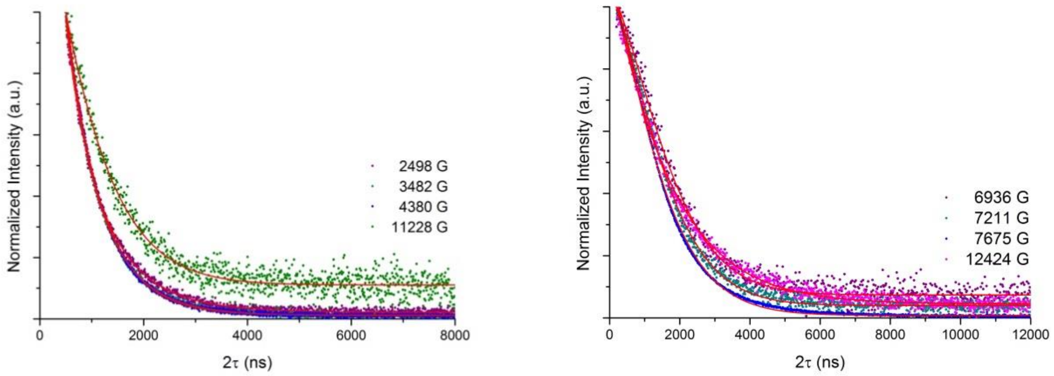

Figure S32. Normalized Hahn echo signal intensities of **1-Ce** and **1-Sm** as a function of the inter-pulse delay 2τ at different magnetic fields at 5 K, measured with π/τ (ns) of (left) 128/500 and (right) 32/200.

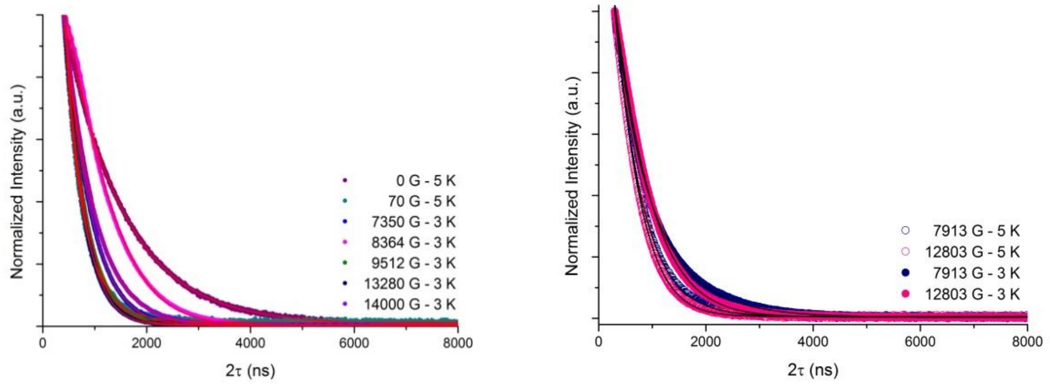

Figure S33. Normalized Hahn echo signal intensities at Q-band of (left) **1-Nd** and (right) **1-Ce** as a function of the inter-pulse delay 2τ at different magnetic fields at 5 K, measured with π/τ (ns) of 40/400.

7.2.3. Spin–lattice Relaxation Time (T_1)

Spin-lattice relaxation time data (Figures S34-S35) were acquired with a standard magnetization inversion recovery sequence, *π–t–π/2–τ–π–τ–echo*,[9] with t_π = 32 ns and τ = 300 ns for X-band and with t_π = 40 ns and τ = 400 ns for Q-band, and variable *t*. The spin-lattice relaxation time constant, T_1, was determined by fitting the experimental data to the following biexponential decay function:

$$Y(t) = Y(0) + Y_1 e^{(-t/T_1)} + Y_{SD} e^{(-t/T_{SD})} \text{ (Equation 3)}$$

where Y_1 and Y_{SD} are the amplitudes, and T_{SD} is the spectral diffusion time constant,[12] giving the results presented in Tables S3-S7. The presence of two decays is commonly attributed to the occurrence of both spectral diffusion (SD) and spin-lattice relaxation (T_1) of which the latter is usually assigned as being the slower process.[13] We notice that the magnetization recovery curves do not reach full saturation below 15 K, indicating that the T_1 spin-lattice relaxation time is very long. Fitting such curves to an exponential model is likely to introduce some inaccuracy in the determination of the T_1 values at these temperatures.

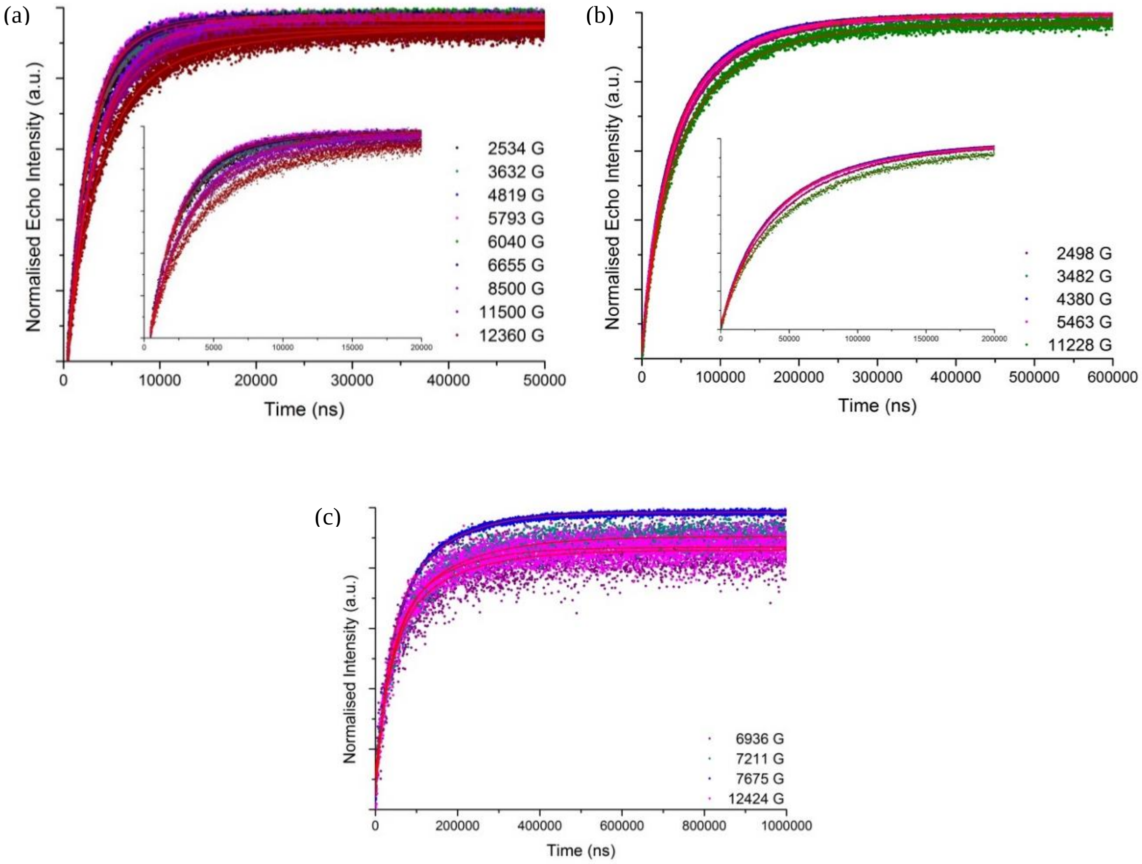

Figure S34. Inversion recovery data for (a) **1-Nd**, (b) **1-Ce** and (c) **1-Sm** measured at 5 K.

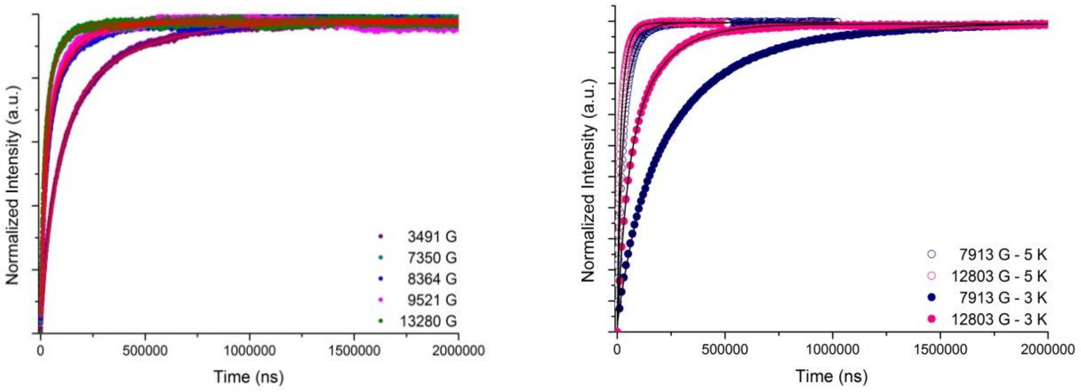

Figure S35. Inversion recovery data at Q-band of (left) **1-Nd** and (right) **1-Ce** measured at 3 and 5 K with π/τ (ns) of 40/400.

Table S3. Extracted spin lattice (T_1) and phase memory (T_m) times and the stretching parameter X for **1-Nd** at 5 K (10 mM tol-hex). T_m was recorded with π/τ (ns) of 32/200,[a] 64/300[b] and 128/400.[c]

Field (G)	T_1 (ns)	T_{SD} (ns)	T_m (ns)	X (eqn1)
**2534**	4084±126	1715±65	254±75[c]	1.031±0.167
**3632**	3660±62	1501±47	313±9[b]<br>246±42[c]	1.139±0.024<br>1.002±0.090
**4819**	4052±84	1706±28	362±6[b]<br>316±29[c]	1.201±0.015<br>1.113±0.065
**5793**	3501±54	1579±30	454±2[a]<br>410±4[b]<br>379±21[c]	1.315±0.007<br>1.232±0.011<br>1.219±0.050
**6040**	4964±46	2181±31	535±1[a]	1.367±0.005
**6655**	4060±79	1756±28	463±2[a]<br>419±5[b]<br>373±24[c]	1.295±0.005<br>1.225±0.013<br>1.138±0.051
**8500**	5771±107	2270±33	520±2[a]	1.281±0.005
**11500**	9864±245	2648±24	670±3[a]	1.303±0.006
**11719**			619±3[a]<br>567±9[b]<br>537±31[c]	1.283±0.008<br>1.248±0.021<br>1.207±0.059
**12360**	12097±332	3215±40	678±5[a]<br>643±13[b]<br>581±40[c]	1.239±0.010<br>1.223±0.025<br>1.166±0.068

Table S4. Extracted spin lattice (T_1) and phase memory (T_m) times and the stretching parameter X for **1-Ce** at 5 K (10 mM tol-hex). T_m was recorded with π/τ (ns) of 128/500.

Field (G)	T_1 (ns)	T_{SD} (ns)	T_m (ns)	X (eqn1)
**2498**	85458±287	28041±146	494±14	0.796±0.011
**3482**	78130±160	24613±74	538±6	0.863±0.005
**4380**	77178±159	23574±80	664±6	0.988±0.006
**5463**	81614±236	25262±110	-	
**11228**	89414±838	27081±469	1077±35	1.195±0.040

Table S5. Extracted spin lattice (T_1) and phase memory (T_m) times and the stretching parameter X for **1-Sm** at 5 K (10 mM tol-hex). T_m measured with π/τ (ns) of 32/200.

Field (G)	T_1 (ns)	T_{SD} (ns)	T_m (ns)	X (eqn1)
**6936**	149245±9106	38586±2934	1882±18	1.457±0.026
**7211**	155023±4426	38261±1436	1732±9	1.394±0.012
**7675**	117638±518	29498±224	1597±4	1.304±0.005
**12424**	125845±4849	30153±1518	1745±16	1.066±0.011

Table S6. Extracted spin lattice (T_1) and phase memory (T_m) times and the stretching parameter X for **1-Nd** at 3 K (10 mM tol-hex) measured at Q-band.

Field (G)	T_1 (ns)	T_{SD} (ns)	T_m (ns)	X (eqn1)
**0**	-	-	1155±2	1.016±0.002
**70**	-	-	235±4	0.8±0.006
**3491**	341755±1634	76010±405	-	-
**7350**	135475±689	28923±163	618±1	1.193±0.001
**8364**	135575±1113	26677±264	1153±3	1.457±0.005
**9521**	87113±831	19079±351	481±1	1.125±0.002
**13280**	65100±733	15655±216	482±1	1.210±0.002
**14000**	-	-	657±1	1.114±0.002

Table S7. Extracted spin lattice (T_1) and phase memory (T_m) times and the stretching parameter X for **1-Ce** at 3 and 5 K (10 mM tol-hex) measured at Q-band.

Field (G)	T_1 (ns)		T_{SD} (ns)		T_m (ns)		X (eqn1)	
	**3 K**	**5 K**	**3 K**	**5 K**	**3 K**	**5K**	**3 K**	**5K**
**7913**	406922 ±3494	49273 ±373	116122 ±2114	15191 ±208	582±3	527±2	0.860 ±0.003	1.021 ±0.003
**12803**	165852 ±2720	28959 ±232	42824 ±1175	8322 ±124	761±2	579±1	1.153 ±0.003	1.205 ±0.002

7.2.4 HYSCORE (Hyperfine sub–level correlation) Measurements

The HYSCORE spectra were recorded at X-band with a four–pulse sequence, $\pi/2–\tau–\pi/2–t_1–\pi–t_2–\pi/2–$*echo*,[9] with pulses $\pi/2$ and π of 16 and 32 ns, respectively, and fixed τ (136, 200 or 400 ns). Times t_1 and t_2 were varied from 100 to 5200 ns in increments of 20 ns. 256 data points were collected in both dimensions. A four-step phase-cycle procedure was used to eliminate unwanted echo contributions. Fourier transformation of the data in both directions yielded 2D (ν_1, ν_2) spectra in which the nuclear cross-peaks (i.e. peaks that correlate nuclear frequencies from opposite spin-manifolds) of the ^{1}H and ^{13}C nuclei appeared in the (+,+) quadrant of the (ν_1, ν_2) map, at separations equivalent with the corresponding hyperfine coupling frequencies (weak coupling regime: $2|\nu_n|>|A|$).[9] The contour line-shape of the cross peaks, and their displacement from the anti-diagonal about the nuclear Larmor frequency (ν_n), relate to the magnitude and anisotropy of the hyperfine couplings, and thus analysis of the HYSCORE spectra allows to determine such parameters.

Spectra modelling using EasySpin assumed that the hyperfine coupling matrix (A) for a given ^{13}C nucleus n is determined by the point dipole (through space) interaction with spin density at the metal ion, given by Equation (4):

$$A^{\text{dip}} = \frac{\mu_0 \beta_e \beta_n}{4\pi h} \frac{3(\bar{\bar{g}} \cdot \text{n})(\tilde{\text{n}} \cdot g_n \bar{\bar{I}}) - \bar{\bar{g}} \cdot g_n \bar{\bar{I}}}{r^3}$$

where g_n is the scalar isotropic nuclear g-value, $\bar{\bar{I}}$ is the identity matrix, r is the distance of the nucleus from the Ln^{3+} ion (in m), n is the Ln-n unit vector expressed in the molecular frame (and ñ is its transpose), β_e and β_n are the electron and nuclear magnetons, h is Plank's constant, and μ_0 is the vacuum permittivity. It is also assumed that g_z lies along the pseudo-C_3 axis (Figure 6), and that the electron spin density is located at the lanthanide ion. A^{dip} is then calculated for each unique carbon position in the Cp^{tt} ligands, using the crystallographic coordinates of the atoms. Simulations considering only A^{dip} reproduce the experimental data satisfactorily for **1-Nd** and **1-Ce** (Figure 6).

Modelling of the ^{1}H HYSCORE data involved a similar approach, including the point dipolar ^{1}H hyperfine (A^{dip}) for the protons of the cyclopentadienyl rings (H^2, H^4, H^5), and all protons of the methyl groups supposed to be close to the Ln(III) ion. Again, good agreement is found with the experimental data for **1-Nd** and **1-Ce** (Figure 6).

7.2.5 HYSCORE Simulation (simple dipolar model)

Table S8. Calculated dipolar interactions of **1-Nd** used for HYSCORE simulations.

**1-Nd**	A_{xx}	A_{xy}	A_{xz}	A_{yx}	A_{yy}	A_{yz}	A_{zx}	A_{zy}	A_{zz}
C1	2.05	0.52	-1.49	0.19	-0.47	-0.08	-0.25	-0.04	-0.12
C2	2.02	1.45	0.025	-0.53	-0.25	0.004	0.004	0.002	-0.22
C3	2.03	0.47	1.52	-0.17	-0.48	0.08	0.255	0.03	-0.12
C4	2.34	-1.58	1.10	-0.58	-0.37	-0.16	0.19	-0.07	-0.22
C5	2.30	-1.52	-1.16	-0.555	-0.38	-0.165	-0.2	0.08	-0.21
H2	2.87	-4.59	0.08	-1.68	0.11	0.02	0.01	0.01	-0.53
H4	2.75	-1.04	0.73	-1.85	-0.03	-0.195	0.12	-0.09	-0.08
H5	2.71	-2.30	-1.76	-1.78	-0.11	0.46	-0.295	0.21	-0.17

Table S9. Calculated dipolar interactions of **1-Ce** used for HYSCORE simulations.

**1-Ce**	A_{xx}	A_{xy}	A_{xz}	A_{yx}	A_{yy}	A_{yz}	A_{zx}	A_{zy}	A_{zz}
C1	-0.05	0.86	1.57	0.51	-0.40	0.66	0.32	0.22	0.15
C2	1.10	0.04	1.81	0.02	-0.75	0.02	0.37	0.006	0.03
C3	-0.02	-0.825	1.49	-0.49	-0.38	-0.62	0.30	-0.21	0.13
C4	-1.39	-0.17	0.64	-0.10	-0.71	-0.655	-0.13	-0.22	0.52
C5	-1.38	0.13	0.50	0.08	-0.69	0.63	0.10	0.21	0.51
H2	4.67	0.16	3.37	0.09	-1.83	0.04	0.68	0.01	-0.32
H4	-3.77	0.40	-0.90	0.24	-1.19	-2.53	-0.18	-0.85	1.17
H5	-3.47	-0.45	-1.09	-0.27	-1.21	2.26	-0.22	0.76	1.11

8. Computational Studies

8.1 Methods

Density functional theory (DFT) optimizations were performed with the PBE[14] functional and Grimme's D3 dispersion correction[15] with Gaussian 16 Rev. C.01.[16] The Stuttgart effective f-in-core pseudopotentials[17] were used for the lanthanide ions and the cc-pVDZ[18] basis set was used to treat the remaining C and H atoms.

Complete active space self-consistent field with spin-orbit coupling (CASSCF-SO)[19,20] calculations with an active space containing all 4f electrons and seven 4f orbitals were performed to compute the magnetic properties of **1-Ce**, **1-Sm**, and **1-Nd**; for **1-Ce** there are two molecules in the unit cell with different metrics so both were calculated. The relativistic atomic natural orbital (ANO-RCC)[21-25] basis sets were used in the CASSCF-SO calculations and the valence orbital treatment was varied based on the distance from the paramagnetic centre: Ln - VTZP; C(Cp), H(Cp), C(tbu1) - VDZP; all other C and H - VDZ. The CASSCF-SO calculations were performed using OpenMolcas version 22.06.[26]

The ^{1}H and ^{13}C HYSCORE simulations of **1-Ce** and **1-Nd** were obtained by calculating relativistic hyperfine coupling constants using DFT-optimized geometries and the above CASSCF-SO methods, feeding into the Hyperion[27] package. The hyperion2easyspin utility was then used to generate EasySpin[28] input files, and the EPR parameters were used to simulate HYSCORE spectra with saffron.[7] Here, using five C(Cp) atoms from one ligand and using three H(Cp) atoms (one H2-, H4- and H5-type atom from one Cp ligand), accurately reproduced the experimental ^{13}C and ^{1}H spectra, respectively.

8.2 Calculated properties of **1-Nd, 1-Ce** and **1-Sm**

Table S10. Energies (in cm^{-1}) and g-values for **1-Nd.**

Energy	g_x	g_y	g_z
0	2.20	2.38	1.08
26.44	0.23	0.02	0.96
126.77	3.74	3.50	0.55
516.77	0.05	0.02	5.48
612.72	2.49	2.57	2.71

Table S11. Crystal field states of **1-Nd.**

	$\vert m_j\rangle$ wavefunction contributions (%)									
**Energy (cm^{-1})**	**-9/2**	**-7/2**	**-5/2**	**-3/2**	**-1/2**	**1/2**	**3/2**	**5/2**	**7/2**	**9/2**
0	0.1	27.3	1.8	0.0	0.0	0.0	0.5	69.8	0.4	0.0
26.44	4.2	0	0.4	53.3	0.5	0.2	34.7	0.1	0.1	6.5
126.77	0.0	0.	0.0	0.7	93.6	5.6	0.1	0.0	0.0	0.0
516.77	12.7	0.0	0.0	9.1	0.1	0.0	1.5	0.0	0.1	76.4
612.72	0.1	56.3	6.9	0.0	0.0	0.0	0.1	20.8	15.8	0.0

Table S12. Energies, g-values and crystal field states of **1-Ce**, molecule 1.

				$\vert m_j\rangle$ wavefunction contributions (%)					
**Energy (cm^{-1})**	**g_x**	**g_y**	**g_z**	**-5/2**	**-3/2**	**-1/2**	**1/2**	**3/2**	**5/2**
0	2.09	3.03	0.71	0.0	0.7	39.6	59.3	0.4	0.0
134.61	0.47	0.44	2.65	0.0	8.1	0.9	0.1	90.7	0.2
859.51	0.16	0.19	3.94	88.4	0.2	0.0	0.0	0.0	11.4

Table S12. cont. Energies, g-values and crystal field states of **1-Ce**, molecule 2.

				$\vert m_j\rangle$ wavefunction contributions (%)					
**Energy (cm^{-1})**	**g_x**	**g_y**	**g_z**	**-5/2**	**-3/2**	**-1/2**	**1/2**	**3/2**	**5/2**
0	2.92	2.21	0.73	0.0	0.1	85.5	13.6	0.7	0.0
105.15	0.36	0.31	2.67	0.3	60.3	0.0	0.8	38.3	0.3
800.55	0.21	0.15	3.95	93.1	0.6	0.0	0.0	0.0	6.3

Table S13. Energies, g-values and crystal field states of **1-Sm**.

				$\|m_j\rangle$ wavefunction contributions (%)					
**Energy (cm^{-1})**	g_x	g_y	g_z	**-5/2**	**-3/2**	**-1/2**	**1/2**	**3/2**	**5/2**
0	0.42	0.42	0.61	0.0	49.4	0.0	0.0	50.6	0.0
400.89	0.003	0.02	0.33	0.0	0.0	58.3	41.7	0.0	0.0
699.23	1.18	1.15	0.85	71.8	0.0	0.0	0.0	0.0	28.2

8.3 Simulated HYSCORE spectra

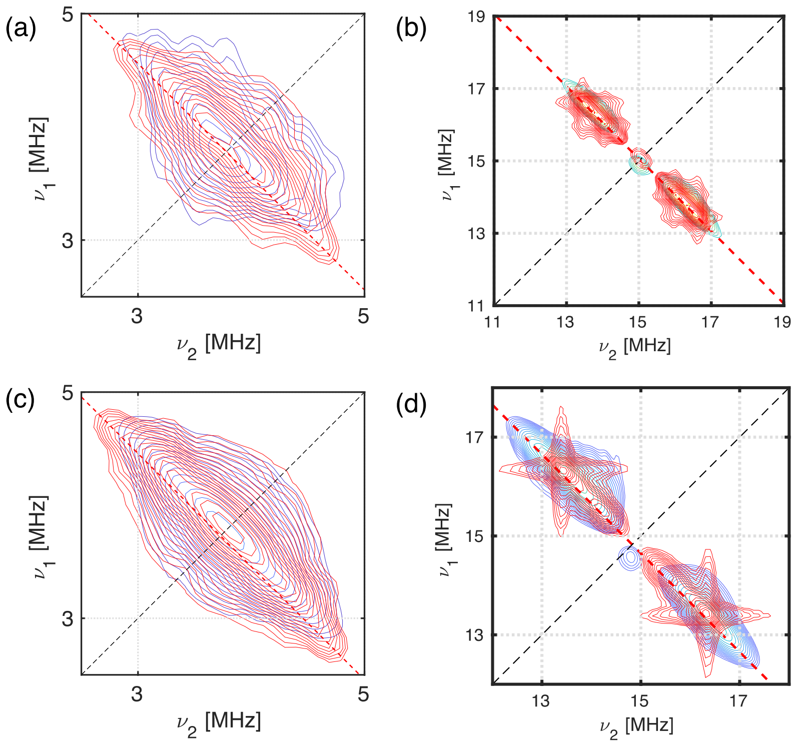

Figure S36. X-Band HYSCORE spectra at a static field of (a), (b) B_0 = 353.0 mT and (c), (d) B_0 = 348.2 mT at 5 K. (a) **1-Nd** ^{13}C region (blue) and CASSCF(3,7)-SO calculated spectrum (red) including C1-C5; (b) **1-Nd** ^{1}H region (blue) with CASSCF(3,7)-SO simulated spectrum (red) with H2, H4, and H5 protons from one ligand; (c) **1-Ce** ^{13}C region (blue) and CASSCF-(1,7)-SO simulated spectrum (red) including C1-C5; (d) **1-Ce** ^{1}H region (blue) and CASSCF(1,7)-SO simulated spectrum (red) with H2, H4, and H5 protons from one ligand.

Table S14. CASSCF-SO hyperfine couplings of **1-Ce** used for HYSCORE simulations, obtained using optimized structure.

**1-Ce**	A_{xx}	A_{xy}	A_{xz}	A_{yx}	A_{yy}	A_{yz}	A_{zx}	A_{zy}	A_{zz}
C1	-0.007	-0.897	0.136	-0.897	-1.847	0.500	0.136	0.500	1.172
C2	-0.007	0.897	-0.136	0.897	-1.847	0.500	-0.136	0.500	1.172
C3	-0.008	0.333	-0.847	0.333	0.844	1.059	-0.847	1.059	-1.531
C4	-0.008	-0.333	0.847	-0.333	0.844	1.059	0.847	1.059	-1.531
C5	-0.007	0.570	0.705	0.570	-0.034	-1.558	0.705	-1.558	-0.648
H2	0.592	0.000	0.000	0.000	1.364	2.054	0.000	2.054	-3.435
H4	-0.843	-2.734	0.164	-2.734	-2.954	-0.889	0.164	-0.889	2.349
H5	-0.843	2.734	-0.164	2.734	-2.954	-0.889	-0.164	-0.889	2.349

Table S15. CASSCF-SO hyperfine couplings of **1-Nd** used for HYSCORE simulations, obtained using optimized structure.

**1-Nd**	A_{xx}	A_{xy}	A_{xz}	A_{yx}	A_{yy}	A_{yz}	A_{zx}	A_{zy}	A_{zz}
C1	0.283	-0.833	0.194	-0.833	-1.578	0.640	0.194	0.640	0.999
C2	0.285	0.831	-0.194	0.831	-1.580	0.641	-0.194	0.641	0.999
C3	0.269	-0.251	0.851	-0.251	0.876	0.785	0.851	0.785	-1.558
C4	0.271	0.250	-0.849	0.250	0.876	0.786	-0.849	0.786	-1.562
C5	0.278	0.633	0.595	0.633	-0.385	-1.442	0.595	-1.442	-0.241
H2	0.904	0.001	0.001	0.001	-1.093	-2.795	0.001	-2.795	-0.729
H4	-0.448	-2.211	1.266	-2.211	-1.422	2.268	1.266	2.268	1.101
H5	-0.442	2.209	-1.265	2.209	-1.427	2.272	-1.265	2.272	1.098

Table S16. Average CASSCF(n,7)-SO Mulliken spin populations of **1-Ce, 1-Sm, and 1-Nd**, obtained using optimized structures.

1-M	M	Cp(C1)	Cp(C2)	Cp(C3)	Cp(C4)	Cp(C5)	% spin density on Cp ligands
**Ce**	0.9988	0.0000	0.0000	0.0001	0.0001	0.0001	0.09
**Nd**	2.9972	0.0002	0.0002	0.0002	0.0002	0.0002	0.10
**Sm**	4.9923	0.0005	0.0005	0.0005	0.0005	0.0005	0.15

9. References

1. *CrysAlisPRO*, version 39.27b; Oxford Diffraction /Agilent Technologies UK Ltd: Yarnton, U.K., 2017.
2. G. M. Sheldrick, *Acta Crystallogr. Sect. A*, 2015, **71**, 3–8.
3. G. M. Sheldrick, *Acta Crystallogr. Sect. C*, 2015, **71**, 3–8.
4. O. V. Dolomanov, L. J. Bourhis, R. J. Gildea, J. A. K. Howard and H. Puschmann, *J. Appl. Crystallogr.* 2009, **42**, 339–341.
5. L. J. Farrugia, *J. Appl. Crystallogr.*, 2012, **45**, 849–854.
6. *POV-Ray. Persistence of Vision Raytracer*; Persistence of Vision Pty. Ltd.: Williamstown, Victoria, Australia, 2013.
7. S. Stoll and R. D. Britt, *Phys. Chem. Chem. Phys*., 2009, **11**, 6614–6625.
8. C. A. P. Goodwin, N. F. Chilton, G. F. Vettese, E. Moreno Pineda, I. F. Crowe, J. W. Ziller, R. E. P. Winpenny, W. J. Evans and D. P. Mills, *Inorg. Chem.*, 2016, **55**, 10057–10067.
9. A. Schweiger and G. Jeschke, *Principles of Pulse Electron Paramagnetic Resonance*, Oxford University Press: Oxford, 2001.
10. C. J. Wedge, G. A. Timco, E. T. Spielberg, R. E. George, F. Tuna, S. Rigby, E. J. L. McInnes, R. E. P. Winpenny, S. J. Blundell and A. Ardavan, *Phys. Rev. Lett.*, 2012, **108**, 107204.
11. A. Formanuik, A.-M. Ariciu, F. Ortu, R. Beekmeyer, A. Kerridge, F. Tuna, E. J. L. Mclnnes and D. P. Mills, *Nat. Chem.*, 2017, **9**, 578–583.
12. K. S. Pedersen, A.-M. Ariciu, S. McAdams, H. Weihe, J. Bendix, F. Tuna and S. Piligkos, *J. Am. Chem. Soc*., 2016, **138**, 5801–5804.
13. K. Bader, M. Winkler and J. van Slageren, *Chem. Commun.*, 2016, **52**, 3623–3626.
14. J. P. Perdew, K. Burke and M. Ernzerhof, *Phys. Rev. Lett.*, 1996, **77**, 3865.
15. S. Grimme, J. Antony, S. Ehrlich and H. A. Krieg, *J. Chem. Phys.*, 2010, **132**, 154104.
16. M. J. Frisch, G. W. Trucks, H. B. Schlegel, G. E. Scuseria, M. A. Robb, J. R. Cheeseman, G. Scalmani, V. Barone, G. A. Petersson, H. Nakatsuji, X. Li, M. Caricato, A. Marenich, J. Bloino, B. G. Janesko, R. Gomperts, B. Mennucci, H. P. Hratchian, J. V Ortiz, A. F. Izmaylov, J. L. Sonnenberg, D. Williams-Young, F. Ding, F. Lipparini, F. Egidi, J. Goings, B. Peng, A. Petrone, T. Henderson, D. Ranasinghe, V. G. Zakrzewski, J. Gao, N. Rega, G. Zheng, W. Liang, M. Hada, M. Ehara, K. Toyota, R. Fukuda, J. Hasegawa, M. Ishida, T. Nakajima, Y. Honda, O. Kitao, H. Nakai, T. Vreven, K. Throssell, J. J. A. Montgomery, J. E. Peralta, F. Ogliaro, M. Bearpark, J. J. Heyd, E. Brothers, K. N. Kudin, V. N. Staroverov, T. Keith, R. Kobayashi, J. Normand, K. Raghavachari, A. Rendell, J. C. Burant, S. S. Iyengar, J. Tomasi, M. Cossi, J. M. Millam, M. Klene, C. Adamo, R. Cammi, J. W. Ochterski, R. L. Martin, K. Morokuma, O. Farkas, J. B. Foresman and D. J. Fox, *Gaussian 09 (revision D.01)*, Gaussian, Inc., 2016.
17. M. Dolg, H. Stoll, A. Savin and H. Preuss, *Theor. Chim. Acta*, 1989, **75**, 173–194.

18. T. H. Dunning Jr., *J. Chem. Phys.*, 1989, **90**, 1007–1023.
19. B. O. Roos, P. R. Taylor and P. E. M. Siegbahn, *Chem. Phys.*, 1980, **48**, 157–173.
20. P. Å. Malmqvist, B. O. Roos and B. Schimmelpfennig, *Chem. Phys. Lett.*, 2002, **357**, 230–240.
21. B. O. Roos, R. Lindh, P. Å. Malmqvist, V. Veryazov and P. O. Widmark, *J. Phys. Chem. A*, 2004, **108**, 2851–2858.
22. B. O. Roos, V. Veryazov and P. O. Widmark, *Theor. Chem. Acc.*, 2004, **111**, 345–351.
23. B. O. Roos, R. Lindh, P. Å. Malmqvist, V. Veryazov and P. O. Widmark, *Chem. Phys. Lett.*, 2005, **409**, 295–299.
24. B. O. Roos, R. Lindh, P. Å. Malmqvist, V. Veryazov and P. O. Widmark, *J. Phys. Chem. A*, 2005, **109**, 6575–6579.
25. B. O. Roos, R. Lindh, P. Å. Malmqvist, V. Veryazov, P. O. Widmark and A. C. Borin, *J. Phys. Chem. A*, 2008, **112**, 11431–11435.
26. F. Aquilante, J. Autschbach, A. Baiardi, S. Battaglia, V. A. Borin, L. F. Chibotaru, I. Conti, L. De Vico, M. Delcey, I. F. Galván, N. Ferré, L. Freitag, M. Garavelli, X. Gong, S. Knecht, E. D. Larsson, R. Lindh, M. Lundberg, P. Å. Malmqvist, A. Nenov, J. Norell, M. Odelius, M. Olivucci, T. B. Pedersen, L. Pedraza-González, Q. M. Phung, K. Pierloot, M. Reiher, I. Schapiro, J. Segarra-Martí, F. Segatta, L. Seijo, S. Sen, D. C. Sergentu, C. J. Stein, L. Ungur, M. Vacher, A. Valentini and V. Veryazov, *J. Chem. Phys.*, 2020, **152**, 214117.
27. L. Birnoschi and N. F. Chilton, *J. Chem. Theor. Comput.*, 2022, **18**, 4719–4732.
28. S. Stoll and A. Schwieger, *J. Magn. Reson.*, 2006, **178**, 42–55.
